# The KH-domain genes *FLK* and *HOS5* integrate flowering and stress responses in *Arabidopsis thaliana*

**DOI:** 10.1093/jxb/eraf286

**Published:** 2025-06-27

**Authors:** Encarnación Rodríguez-Cazorla, Juan-José Ripoll, Héctor Candela, Almudena Aranda-Martínez, Ernesto Zavala-González, José-María García-Mina, Ángel-María Zamarreño, Antonio Martínez-Laborda, Antonio Vera

**Affiliations:** Area de Genética, Universidad Miguel Hernández, Campus de Sant Joan, San Juan de Alicante 03550, Spain; Area de Genética, Universidad Miguel Hernández, Campus de Sant Joan, San Juan de Alicante 03550, Spain; Instituto de Bioingeniería, Universidad Miguel Hernández, Campus de Elche, Elche 03202, Spain; Department of Microorganisms, Atlántica Agrícola S.A. Polígono Industrial El Rubial, Villena, Alicante 03400, Spain; Department of Microorganisms, Atlántica Agrícola S.A. Polígono Industrial El Rubial, Villena, Alicante 03400, Spain; Instituto de Biodiversidad y Medioambiente BIOMA, Universidad de Navarra, Irunlarrea 1, Pamplona 31008, Spain; Instituto de Biodiversidad y Medioambiente BIOMA, Universidad de Navarra, Irunlarrea 1, Pamplona 31008, Spain; Area de Genética, Universidad Miguel Hernández, Campus de Sant Joan, San Juan de Alicante 03550, Spain; Area de Genética, Universidad Miguel Hernández, Campus de Sant Joan, San Juan de Alicante 03550, Spain; University of Auckland, New Zealand

**Keywords:** *Arabidopsis thaliana*, *FLC*, *FLK*, flowering time, *HOS5*, KH-domain gene, RNA regulation, stress response

## Abstract

Plant reproductive success largely relies on flowering under favorable conditions. However, stress factors have forced plants to acquire adaptive strategies to coordinate floral timing and stress responses through key genetic regulators. RNA-binding proteins with K-homology (KH) domains are emerging as versatile regulators of an increasing number of plant developmental processes, including flowering and stress acclimation. In *Arabidopsis thaliana*, the KH-domain genes *FLOWERING LOCUS K* (*FLK*) and *HIGH OSMOTIC STRESS GENE EXPRESSION 5* (*HOS5*) are associated with transcription and co-transcriptional operations. *FLK* facilitates floral transition by repressing *FLOWERING LOCUS C* (*FLC*), the central flowering inhibitor, while both KH-domain genes have been shown to be involved in abiotic stress and pathogen defense. Our genetic and molecular data identify HOS5 as a novel flowering regulator that acts in concert with FLK to repress *FLC*. Our transcriptomic results reveal that, in addition, *FLK* and *HOS5* cooperatively repress numerous stress-responsive genes. Consistent with this, *flk hos5* double mutant plants exhibit elevated levels of stress-induced gene activities and enhanced resistance to abiotic stress and pathogenic fungi. The coordinated repression of *FLC* and stress-induced genes, together with the interaction at the molecular level, suggests that *FLK* and *HOS5* participate in a co-transcriptional regulatory hub key for orchestrating flowering time and environmental adaptation responses. This study aims to better define the role of KH-domain genes and provides candidates for their exploitation in crop biotechnology.

## Teaser

The Arabidopsis K-homology genes *FLK* and *HOS5* interact to coordinate rapid co-transcriptional regulation of flowering and stress pathways to adapt plant responses to endogenous and environmental changes.

## Introduction

To initiate flowering under optimal conditions, plants have evolved a sophisticated network of regulatory pathways that sense environmental and endogenous cues (photoperiod, temperature, and developmental phase), that finally converge on floral integrators, triggering the formation of flowers (Kinoshita and Richter, 2020; Freytes *et al.*, 2021; Quiroz *et al.*, 2021). In the reference plant *Arabidopsis thaliana* (Arabidopsis hereafter), the autonomous pathway (AP) genes promote flowering independently of daylength by repressing the central flowering inhibitor *FLOWERING LOCUS C* (*FLC*) (Wu *et al.*, 2020). *FLC* encodes a MADS-domain transcription factor that prevents precocious flowering by directly repressing floral integrators such as *FLOWERING LOCUS T* (*FT*; the florigen) and *SUPPRESSOR OF OVEREXPRESSION OF CONSTANS1* (*SOC1*) (Michaels and Amasino, 1999; Li *et al.*, 2008; Jang *et al.*, 2009).

In addition to daily and seasonal ambient fluctuations, plants are often exposed to biotic (pathogenic microorganisms) or abiotic stress conditions (including drought, salinity, or extreme temperatures), which are further exacerbated by global climate change, compromising survival and reproduction (Fichman and Mittler, 2020). To neutralize these effects, plants have developed complex defense mechanisms that balance stress tolerance and growth, ultimately affecting yield (Park *et al.*, 2016; Zhang *et al.*, 2020b). One significant response to stress is the modification of flowering time through an intricate genetic and molecular network connecting both processes (Kazan and Lyons, 2016). For example, salinity usually delays flowering whereas drought can accelerate it. Indeed, stress and flowering pathways ultimately converge on common endogenous floral regulators (Kazan and Lyons, 2016). For instance, short periods of cold stress delay flowering by activating *FLC* (Jung *et al.*, 2013), and the AP genes *FPA* and *FLOWERING LOCUS D* (*FLD*) negatively regulate resistance against the bacterial pathogen *Pseudomonas syringae* (Lyons *et al.*, 2013; Singh *et al.*, 2013), whereas several flowering genes, including *FLC*, affect tolerance to cucumber mosaic virus (Shukla *et al.*, 2022). Therefore, dissecting the genetic mechanisms underlying the crosstalk between flowering and stress pathways is crucial for understanding the adaptation of floral timing to challenging environmental conditions.

As sessile organisms, plants must respond to environmental variations through rapid changes in gene regulation. Transcriptional reprogramming and associated co-transcriptional pre-mRNA processing, including 5′ capping, splicing, and 3′ cleavage/polyadenylation, are major determinants of gene expression, and also generate multiple isoforms that increase developmental flexibility and adaptive responses (Ambrosone *et al.*, 2012; Marquardt *et al.*, 2023). RNA-binding proteins are crucial to accomplish these functions (Ambrosone *et al.*, 2012; Bentley, 2014; Rodríguez-Cazorla *et al.*, 2015, 2018; Marquardt *et al.*, 2023; Shine *et al.*, 2024). *FLOWERING LOCUS K* (*FLK*) encodes an RNA-binding protein with K-homology (KH) domains that, as an AP member, promotes flowering via *FLC* suppression (Lim *et al.*, 2004; Mockler *et al.*, 2004). The KH-domain, originally identified in the human heterogeneous nuclear ribonucleoprotein K (hnRNPK; Siomi *et al.*, 1993), is an ancient motif important for binding to ssDNA/RNA, and provides a structural basis for protein–protein interactions (Makeyev and Liebhaber, 2002; Nicastro *et al.*, 2015). Thus, proteins with KH-domains are involved in all levels of gene regulation, and disruption of KH-domain genes is linked to severe human disorders (Lewis *et al.*, 2000; Makeyev and Liebhaber, 2002; Hasan and Brady, 2024). The structure of FLK, harboring three KH motifs, closely resembles that of metazoan poly(rC)-binding proteins (PCBPs), a functionally versatile family of proteins that includes hnRNPK (Lim *et al.*, 2004; Zhao *et al.*, 2022). Interestingly, a recent study identified FLK as an *N*^6^-methyladenosine (m^6^A) reader that represses *FLC* by reducing mRNA stability and splicing (Amara *et al.*, 2023). Additionally, previous evidence also suggested a transcriptional role for *FLK* via chromatin modulation (Veley and Michaels, 2008).

During flower morphogenesis, *FLK* acts in concert with two other KH genes, *PEPPER* (*PEP*) and *HUA ENHANCER4* (*HEN4*), to secure the correct expression of the floral master regulator *AGAMOUS* (*AG*), a MADS-box-encoding gene similar to *FLC* (Cheng *et al.*, 2003; Rodríguez-Cazorla *et al.*, 2015). FLK, PEP, and HEN4 interact at the protein level, suggesting that they participate in the same complexes to regulate their targets co-transcriptionally (Rodríguez-Cazorla *et al.*, 2015). However, *PEP* and *HEN4* promote *FLC* expression, thus antagonizing *FLK* during flowering regulation (Ripoll *et al.*, 2009; Ortuño-Miquel *et al.*, 2019). This suggests that the function and/or composition of common RNP assemblies is dynamic and complex, and most probably involving as yet unknown additional partners.

In addition to flowering and floral morphogenesis, *FLK* has been recently linked to pathogen defense and salicylic acid (SA) homeostasis (Fabian *et al.*, 2023), making this gene an appealing candidate to coordinate stress responses and adaptation of reproductive development. However, the mechanisms by which *FLK* links both operations remain unclear. The identification of additional *FLK*-interacting factors might reveal additional insights into our understanding about these processes. The gene *HIGH OSMOTIC STRESS GENE EXPRESSION 5* (*HOS5*), also known as *SHINY1* (*SHI1*), *REGULATOR OF CBF GENE EXPRESSION3* (*RCF3*), or *ENHANCED STRESS RESPONSE1* (*ESR1*), encodes another KH-domain protein involved in abiotic stress and pathogen resistance (Xiong *et al.*, 1999; Chen *et al.*, 2013; Guan *et al.*, 2013; Jeong *et al.*, 2013; Jiang *et al.*, 2013; Karlsson *et al.*, 2015; Thatcher *et al.*, 2015). HOS5 has been proposed to regulate splicing, and repress transcription of stress-inducible genes by preventing mRNA capping, and thus the transition to transcript elongation (Chen *et al.*, 2013; Jiang *et al.*, 2013). Interestingly, the FLK-binding partner PEP was found to interact with the phosphatase CPL1, a critical regulator of the C-terminal domain (CTD) of RNA polymerase II (Rodríguez-Cazorla *et al.*, 2018). HOS5 was also identified as a CPL1 interactor via their KH-domains, similar to those of PEP and FLK (Chen *et al.*, 2013; Jeong *et al.*, 2013; Jiang *et al.*, 2013; Karlsson *et al.*, 2015). In addition, the *hos5* mutant displays altered polyadenylation site selection and intron retention under particular conditions (Chen *et al.*, 2013; Jiang *et al.*, 2013). Thus, we decided to explore the functional connection between *HOS5* and *FLK* gene activities.

To better delineate the role of *FLK* in flowering adaptation and stress, we have explored its relationship with *HOS5*. Strong genetic interactions provide evidence that both genes act in concert to repress *FLC* expression, revealing *HOS5* as a novel flowering regulator. We also show that *FLK* and *HOS5* co-regulate numerous genes involved in stress responses. In line with this, *flk hos5* double mutants show up-regulation of numerous ‘stress genes’, elevated levels of the defense hormones jasmonic acid (JA) and SA, and higher tolerance to abiotic stressors and pathogenic fungi. Our genetic and molecular data support a model in which *FLK* and *HOS5* directly cooperate as part of a regulatory module that integrates plant developmental outputs (flowering) and environmental (stress) adaptive responses, a view reinforced by the ability of FLK and HOS5 to associate *in planta*. We also discuss possible mechanisms by which *FLK* and *HOS5* interact to regulate mRNA expression of *FLC* and additional gene targets. This study further expands our knowledge on the underlying molecular mechanisms governing flowering and stress response coordination, assists in better delineating the role of KH-domain genes in Arabidopsis, and provides candidates for their exploitation in crop biotechnological strategies.

## Materials and methods

### Plant material

All strains in this work were in the Arabidopsis Columbia (Col-0) accession: *flk-2* (Mockler *et al.*, 2004), *hos5-2* (Chen *et al.*, 2013), *hos5-5* (SALK_013918, this work), and *flc-3* (Michaels and Amasino, 1999). Information about molecular genotyping and primers used in this work can be found in Supplementary Table S1.

### Standard growth conditions and flowering time measurements

Seeds were surface-sterilized, stratified for 2 d at 4 °C and grown on half Murashige and Skoog (MS) plates at 21 °C under long-day (16–8 h) or short-day (8–16 h) regimes (130 mol m^−2^ s^−1^ generated by Sylvania standard F65W cool white light fluorescent tubes), as previously described (Ripoll *et al.*, 2009; Zavala-Gonzalez *et al.*, 2017). Fourteen-day-old seedlings were transplanted to individual pots with soil, and inspected daily for flowering (days and rosette leaves at bolting). Unless otherwise indicated, 30 plants per genotype were analyzed in a single assay, and every experiment was carried out three times.

### Germination and growth under salt and methyl viologen

Seeds were sown on medium supplemented with varying concentrations of NaCl, under long-day conditions. Germination was determined counting radicle emergence under a dissecting microscope. For methyl viologen (MV; Paraquat) treatments, seeds were plated onto medium with 0.5 μM or 1 μM MV (Sigma-Aldrich), and seedlings with green, fully emerged cotyledons were counted. A minimum of 100 seeds per replicate were scrutinized for each genotype under analysis. Standard deviation (SD) was calculated from three independent experiments, except for growth at 50 mM NaCl (SD calculated from two replicates with 12 plants per genotype).

### Methyl-jasmonate root inhibition assays

Seeds were grown on vertically oriented control plates or supplemented with 50 µM methyl-jasmonate (MeJA). Seven-day-old plants were photographed, and primary root length was determined using Image J software. Three independent experiments were conducted with 20 plants per genotype in each assay.

### Quantitative reverse transcription–PCR

All RNA extractions were conducted at Zeitgeber time (ZT) 3 (h). For quantitative reverse transcription–PCR (qRT–PCR) procedures, 5 μg of total RNA was extracted from 12-day-old rosettes, treated with DNase I, and used for cDNA synthesis with an oligo(dT) primer and RevertAid Premium Reverse Transcriptase (Thermo Fisher, Waltham, MA, USA) following the manufacturer's instructions, as previously reported (Rodríguez-Cazorla *et al.*, 2020). Subsequently, for each qRT–PCR, 0.5 μl of the cDNA was used as template. Relative changes in gene expression levels were determined using the LightCycler 1.5 system (Roche Diagnostics, Basel, Switzerland) with the Maxima SYBR Green qPCR master mix (Thermo Fisher) according to the manufacturer. RNA levels were normalized to the constitutively expressed genes *OTC* (*ORNITINE TRANSCARBAMYLASE*) and *ACT2* (*ACTIN2*), and the corresponding wild-type levels, as reported previously (Rodríguez-Cazorla *et al.*, 2015). For each experiment, three biological replicates were performed, with three technical replicates each. Splicing efficiency was determined, for each intron examined, as the level of spliced transcript normalized to the amount of unspliced transcript, and represented as the fold change (FC) over Col-0 values from three independent assays.

### RNA sequencing and bioinformatics analysis

Total RNA was extracted (Rodríguez-Cazorla *et al.*, 2015) from pooled 12-day-old rosettes. A 1 μg aliquot of RNA per sample was used for cDNA library construction with the TruSeq Stranded mRNA LT Sample Prep Kit for Illumina^®^ (NEB, USA). The resulting fragments were sequenced on the lllumina Hiseq 2500 platform, using 151 bp paired-end reads, at Macrogen (South Korea). Paired-end reads were first processed using Trimmomatic v. 0.39 (Bolger *et al.*, 2014) with options ILLUMINACLIP:TruSeq3-PE.fa:2:30:10:2:keepBothReads LEADING:3 TRAILING:3 MINLEN:36. The reads were then aligned to the TAIR10 version of the *A. thaliana* genome sequence (https://www.arabidopsis.org/) using Hisat 2 version 2.2.1 (Kim *et al.*, 2019), considering the strandness of the reads (with option --rna-strandness RF) and discarding all discordant read mappings (with options no-discordant and no-mixed). Transcript levels were quantified for the ARAPORT11 gene models using the cuffdiff program of the Cufflinks version 2.2.1 package (Trapnell *et al.*, 2013), selecting fr-firststrand as the library type. HTSeq-count (version 2.0.5; Anders *et al.*, 2015) was used to count reads aligned to introns, with the following parameters: -f bam -r pos -s reverse --nonunique all -t intron -i gene_id. The resulting counts were analyzed using the DESeq2 package (version 1.38.3; Love *et al.*, 2014) in R (version 4.2.2). Introns with an adjusted *P*-value <0.05 and an absolute log2 FC >1 were considered significantly differentially expressed. Three biological replicates were used for each genotype. The resulting read alignments, supplied as files in BAM format, were visualized using Integrative Genomics Viewer (IGV) software (Thorvaldsdottir *et al.*, 2013). Identification of over-represented Gene Ontology (GO) terms was performed as implemented by the Panther classification system in the Gene Ontology website (http://geneontology.org/) using a selected set of genes (including those marked ‘OK’ by Cufflinks) as the customized annotated reference, as previously described (Muñoz-Nortes *et al.*, 2017). Fisher’s exact test was used as the test type, with Bonferroni correction for multiple testing.

### Bimolecular fluorescence complementation and yeast two-hybrid assays

Bimolecular fluorescence complementation (BiFC) and yeast two-hybrid (Y2H) experiments were performed as previously described (Ripoll *et al.*, 2015; Guan *et al.*, 2017; Rodríguez-Cazorla *et al.*, 2018). Briefly, for Y2H assays, the LexA-inducible system was applied. The cDNA PCR amplicons for *HOS5*, *CPL1*, and *FLK* genes were generated using the corresponding primers (Supplementary Table S1) and cloned into the pB42AD (+Trp) and pGilda (+His) vectors via the Gibson DNA assembly procedure (Gibson, 2011). The integrity of the resulting pGilda and pB42AD constructs was checked by sequencing. The yeast strain EGY48 (−Ura) was co-transformed with the corresponding combinations of pGilda and pB42AD constructs. Empty vectors were used as negative controls. Positive colonies were selected on solid media (−Ura, −His, −Trp+glucose). Induction for testing protein–protein association was assayed by growing the resulting yeast strains on plates in the presence of galactose and raffinose (DB Falcon). X-gal (SIGMA) was used for colorimetric assays. For BiFC, the corresponding coding sequences were amplified from their respective cDNAs using the proofreading Phusion (New England Biolabs, Inc.) polymerase (Supplementary Table S1) and cloned into pBJ36-SPYNE and/or pBJ36-SPYCE plasmids, containing N-terminal (nt) and C-terminal (ct) halves of the yellow fluorescent protein (YFP), respectively (YFPnt and YFPct). The resulting 35S::SPYNE and 35S::SPYCE cassettes were sequenced and then cloned into the T-DNA binary vectors pGreen0229 and pGreen0179 (Hellens *et al.*, 2000), respectively. Transformed AGL-0 *Agrobacterium tumefaciens* cells were used to infect *Nicotiana benthamiana* leaves. YFP reconstituted fluorescence was visualized 72 h after inoculation under a Nikon Eclipse TE2000-U epifluorescence microscope. As negative controls, *Nicotiana* leaves were co-infiltrated with the corresponding recombinant YFPct construct and the empty YFPnt version.

### Quantification of plant hormones

Measurements of JA and SA were carried out as in Zavala-Gonzalez *et al.* (2017), according to Seo *et al.* (2011). For every measurement, 100 mg of plant material (12-day-old pooled rosettes) were freeze-dried and suspended in 80% methanol–1% acetic acid containing internal standards and mixed by shaking for 1 h at 4 °C. The extract was kept at −20 °C overnight and then centrifuged, and the supernatant was dried in a vacuum evaporator. The dry residue was dissolved in 1% acetic acid and passed through an Oasis HLB (reverse phase) column as described in Seo *et al.* (2011). For quantification, the dried eluate was dissolved in 5% acetonitrile–1% acetic acid, and the hormones were separated using an autosampler and reverse-phase ultraperformance hydrophilic chromatography (2.6 Å Accucore RP-MS column, 50 mm length×2.1 mm internal diameter; ThermoFisher Scientific) with a 5–50% acetonitrile gradient containing 0.05% acetic acid, at 400 μl min^–1^ for 14 min. The hormones were analyzed with a Q-Exactive mass spectrometer (Orbitrap detector; ThermoFisher Scientific) by targeted selected ion monitoring. The concentrations of hormones in the extracts were determined using calibration curves. At least 20 plants per sample were used and the experiment was carried out three times. The jasmonoyl-isoleucine (JA-Ile) content was determined as described in Olaetxea *et al.* (2024). Briefly, plant material was freeze-dried as above and suspended in a methanol–formic acid mixture containing 2.5 mM sodium diethyldithiocarbamate and the internal standard. After two steps of shaking and centrifugation, the extraction was repeated with the initial extractant, and the combined solution was evaporated. The residue was re-dissolved in methanol/acetic acid, centrifuged, and transferred to an injection vial for chromatographic separation and MS.

### Fungal inoculation

Fungal strains *Botrytis cinerea* (BC03, CECT No. 20973, IRTA Institute, Spain) and *Fusarium oxysporum* (EAN 350, CECT No. 2154) were maintained in Potato Dextrose Agar (PDA), and subcultured monthly. Conidia were obtained from 25-day-old colonies on PDA plates using 0.02% Tween-20. Resulting conidia suspensions were filtered with glass wool, counted using a hemocytometer, and adjusted to 10^5^ spores ml^–1^. A drop (10 µl) of spore solution was applied on the top of each 2-week-old plant grown on agar plates (long day), and photographed 15 d after inoculation. Forty plants per genotype were examined, and the experiments were repeated three times.

### Statistics

Data were subjected to ANOVA to determine significant differences among genotypes (*P<0.05; **P<0.01; ***P<0.001). SD was calculated in Microsoft EXCEL from aggregate data from independent experiments. For qRT–PCR experiments and germination under salt stress, relative expression was calculated according to Pfaffl *et al.* (2002), and statistical significance was estimated by Student’s *t*-test (*P<0.05; **P<0.01; ***P<0.001).

## Results

### The FLK and HOS5 proteins interact *in planta*

**Fig. 1.**
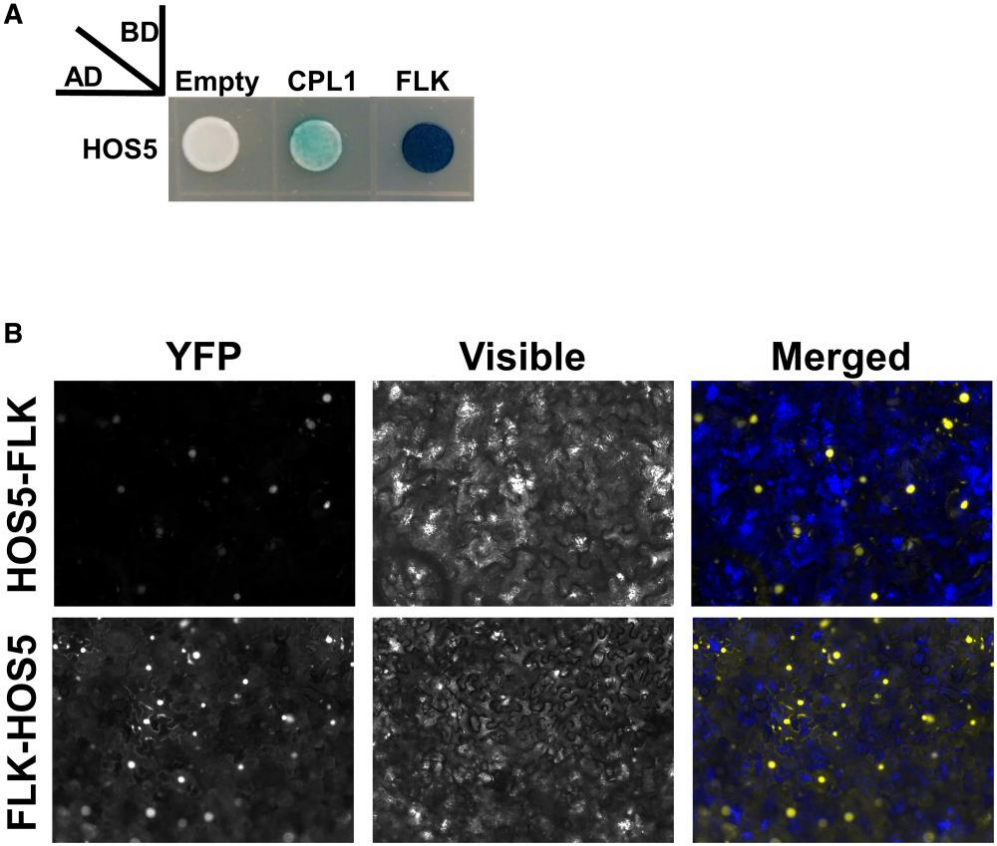
Physical interaction of FLK and HOS5. (A) FLK–HOS5 interaction in yeast two-hybrid assays. A colorimetric assay (X-gal, blue colonies) was used to monitor interaction. CPL1–HOS5 interaction was previously reported and used as a positive control (Chen *et al.*, 2013; Jeong *et al.*, 2013; Jiang *et al.*, 2013). (B) BiFC visualization of protein dimerization (yellow fluorescence) in *Nicotiana benthamiana* leaf cells agroinfiltrated with constructs encoding HOS5 and FLK fusion proteins. In each test, the first protein was fused to the C-terminal fragment of YFP (YFPct), and the second protein to the N-terminal portion (YFPnt). In the merged (visible+YFP fluorescence) picture, yellow nuclei are seen on a blue background used to increase contrast.

*FLK* and *HOS5* encode KH-domain polypeptides that regulate transcription and co-transcriptional RNA processing of their target genes (Jeong *et al.*, 2013; Jiang *et al.*, 2013; Rodríguez-Cazorla *et al.*, 2018; Amara *et al.*, 2023). RNA-binding proteins, including KH-domain proteins, often participate in multimeric RNP complexes to perform their regulatory functions (Shine *et al.*, 2024). We previously identified other KH-domain protein partners of FLK (Rodríguez-Cazorla *et al.*, 2015) and, interestingly, FLK and HOS5 were shown to co-purify with FPA and other 3′-end processing factors (Parker *et al.*, 2021). Therefore, we sought to determine whether FLK and HOS5 could associate. Indeed, our Y2H assay supports FLK–HOS5 physical interaction (Fig. 1A; Supplementary Fig. S1A). To substantiate this result, we performed *in planta* BiFC and further validated the association between FLK and HOS5 in leaf cell nuclei (Fig. 1B; Supplementary Fig. S1B), consistent with the localization of the individual proteins (Mockler *et al.*, 2004; Jiang *et al.*, 2013). These findings support the notion that FLK and HOS5 interact with each other.

### *HOS5* interacts with *FLK* to coordinately repress *FLC* and promote flowering

**Fig. 2.**
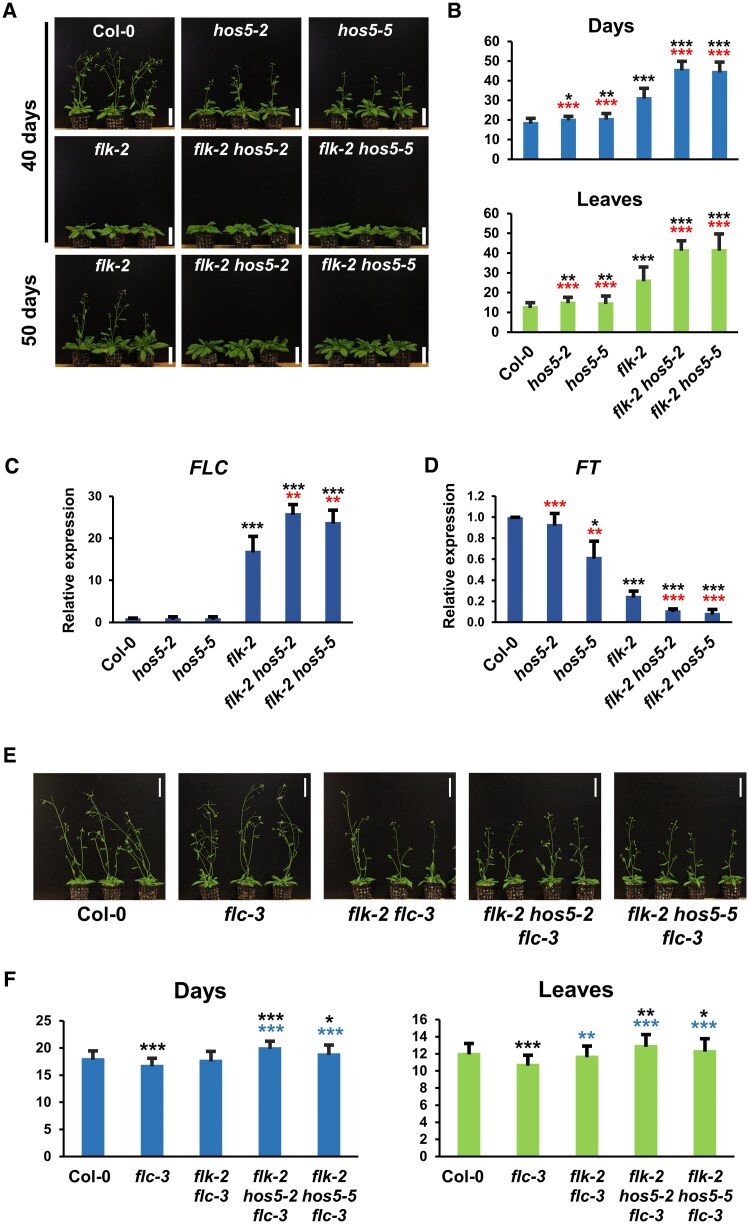
*FLK* and *HOS5* interact to promote flowering via *FLC* repression. (A) Representative 40- and 50-day-old Col-0 (wild type) and mutant plants. (B) Flowering time of Col-0 and mutant plants (number of days or rosette leaves at bolting). (C, D) Relative expression of *FLC* (C) and *FT* (D) monitored by qRT–PCR. Data correspond to three biological replicates with three technical replicates each. Bars represent the mean ±SD. (E) Representative 37-day-old Col-0 and mutant plants harboring *flc-3*. (F) Flowering time at bolting for each of the corresponding backgrounds. For flowering assays (B, F), bars indicate the mean ±SD from three independent experiments, with at least 30 plants per genotype each. For flowering and/or qRT–PCR, black, red, and blue asterisks indicate significant differences with respect to Col-0, *flk-2*, and *flc-3*, respectively (*P<0.05; **P<0.01; ***P<0.001). Scale bars, 5 cm.

To explore the connections between *FLK* and *HOS5*, we generated double mutants using the null T-DNA alleles *hos5-2*, *hos5-5* (Supplementary Fig. S2), and *flk-2* (Mockler *et al.*, 2004). None of the single or double mutants showed any conspicuous morphological defect when compared with wild-type Col-0 plants (Supplementary Fig. S3). However, under long-day conditions, *hos5* mutants flowered slightly later than Col-0, whereas, as previously reported (Lim *et al.*, 2004), *flk-2* plants flowered much later (Fig. 2A, B). Strikingly, flowering was dramatically delayed in *flk-2 hos5* double mutants with respect to *flk-2* (Fig. 2A, B). On average, *flk-2* plants flowered after 30 d and 25 leaves, whereas *flk-2 hos5* double mutants required >45 d and 40 leaves to bolt (Fig. 2B), revealing a very strong interaction between *FLK* and *HOS5*. Under short-day conditions, *flk-2 hos5-5* plants also flowered significantly later than *flk-2*, the difference being even more pronounced than under long days (Supplementary Fig. S4). This strong genetic interaction uncovers a new role for *HOS5*, in concert with *FLK*, in flowering time regulation.

*flk* plants flower late due to *FLC* overexpression (Lim *et al.*, 2004). *FLC* presents four isoforms, with variant 1 being, by far, the most abundant (Cai *et al.*, 2023). We monitored, by qRT–PCR, *FLC* expression as the amount of transcript corresponding to correctly spliced intron 1, common to all four isoforms. In *hos5* mutants, *FLC* mRNA levels were largely similar to those of Col-0. However, in *flk-2 hos5* plants, *FLC* abundance was significantly higher than that in *flk-2* (Fig. 2C). These results closely correlate with the observed delay in flowering time (Fig. 2B, C), and suggest that *FLC* misexpression is the likely cause of this phenotype. Consistent with this, the expression of integrator genes repressed by *FLC* was significantly down-regulated in *flk-2* and *flk-2 hos5* mutants (Fig. 2D; Supplementary Fig. S5). These findings were consistently observed in both combinations of *flk-2 hos5* double mutants (Fig. 2; Supplementary Fig. S5), indicating that *hos5-2* and *hos5-5* are equivalent null alleles. We therefore adopted *hos5-5* as the reference hereafter.

To confirm the direct involvement of *FLC* in the flowering phenotypes, we introduced the *flc-3* null allele (Michaels and Amasino, 1999) into both *flk-2* and *flk-2 hos5* plants. In the resulting backgrounds, flowering delay was abolished (Fig. 2E, F), providing genetic evidence that the late-flowering phenotypes of *flk-2 hos5* mutants result from *FLC* up-regulation. However, a mild but significant delay in the *flk-2 hos5 flc-3* mutants, as compared with Col-0 and *flc-3* individuals, suggests the existence of minor *FLC*-independent effects (Fig. 2F), probably due to modest up-regulation in this background of other *FLC*-clade members, such as *MADS AFFECTING FLOWERING 4* (*MAF4*) and *MAF5* (Ratcliffe *et al.*, 2003; Supplementary Fig. S6).

### High levels of *FLC* expression mediate the germination vigor of *flk hos5* seeds

*FLC* inhibits flowering, but positively regulates other developmental processes, such as the germination transition, making its expression a pleiotropic trait of significant adaptive relevance. *FLC* enhances germination efficiency by modulating gene activities that reduce the germination-repressive hormone abscisic acid and trigger the germination-inductive gibberellins (Chiang *et al.*, 2009). Consistent with this, high *FLC*-expressing strains, such as *flk hos5* double mutants, often exhibit robust germination. Therefore, to further support our observations on flowering time, we tested wild-type and mutant germination under salt stress.

**Fig. 3.**
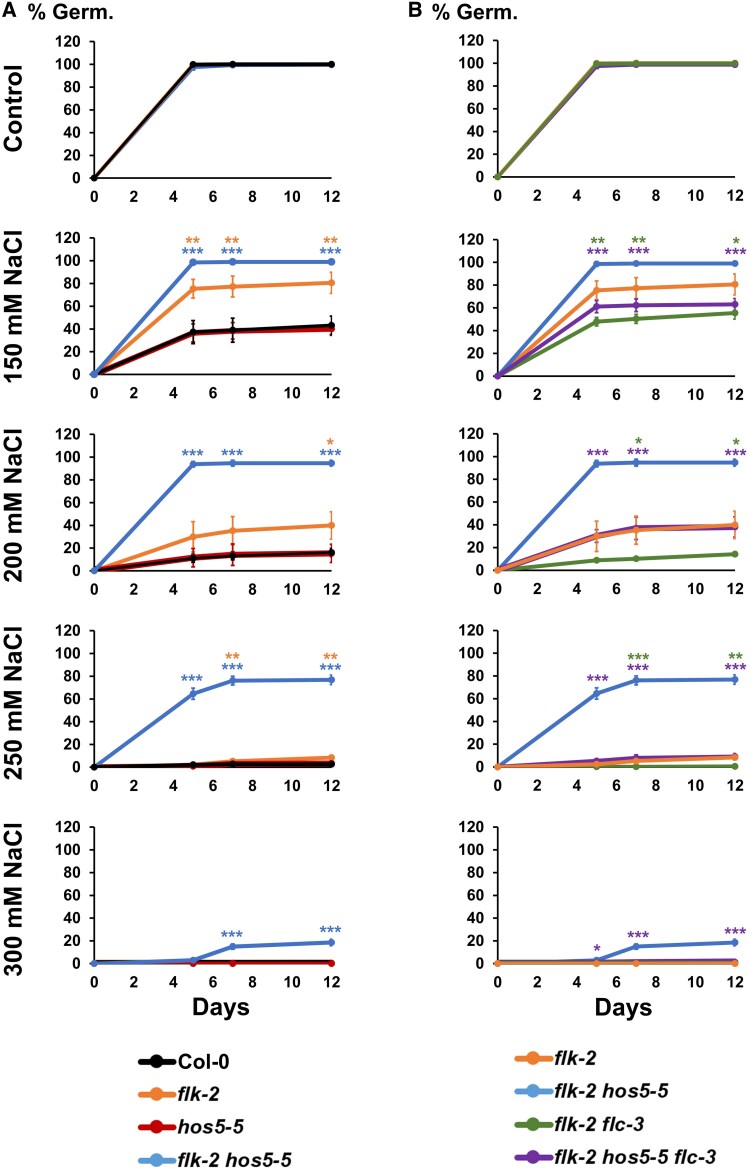
*FLC* mediates *flk hos5* germination vigor under salt stress. (A) Percentage of germination in the wild-type Col-0, and *flk-2, hos5-5*, and *flk-2 hos5-5* mutant backgrounds. (B) Percentage of germination in the *flk-2 flc-3* and *flk-2 hos5-5 flc-3* mutant backgrounds. For a better comparison, and to underscore the relevance of *FLC* for their elevated germination rates, the same *flk-2* and *flk-2 hos5-5* data shown in (A) are also included in (B). The appearance of visible radicles was used as a morphological marker for germination. Three independent measurements, with no less than 100 seeds, were averaged. Bars indicate the mean ±SD. In (A), orange and blue asterisks denote significant differences of *flk-2* and *flk-2 hos5-5* with respect to Col-0. In (B), green asterisks denote significant differences between *flk-2 flc-3* and *flk-2*, whereas purple asterisks denote significant differences between *flk-2 hos5-5 flc-3* and *flk-2 hos5-5* (*P<0.05; **P<0.01; ***P<0.001).

We scored the percentage of germination on agar medium with increasing NaCl concentrations. In control plates, all genotypes rapidly reached 100% germination rates, and salinity correlated with lower germination percentages (Fig. 3A). Under salt stress, Col-0 and *hos5-5* exhibited similar poor germination rates, whereas *flk-2* and *flk-2 hos5-5* seeds germinated more vigorously. Strikingly, at 300 mM NaCl, *flk-2 hos5-5* seeds exhibited a 20% germination rate, while it was completely inhibited for the rest of the genotypes assayed (Fig. 3A).

Germination rates of the *flk-2* and *flk-2 hos5-5* genotypes are consistent with our hypothesis, and nicely correlated with higher *FLC* mRNA levels observed during seedling development. Therefore, to further substantiate these observations, we studied *flk-2 flc-3* and *flk-2 hos5-5 flc-3* germination under salt stress. The lack of *FLC* greatly reduced the ability to germinate in saline medium. In *flk-2 flc-3*, germination rates plummeted to wild-type values (Fig. 3B). Similarly, the *flk-2 hos5-5 flc-3* seed germination rates were much lower than those of *flk-2 hos5-5*. However, *flk-2 hos5-5 flc-3* triple mutants still germinated slightly better than Col-0 seeds (Fig. 3B). This suggests that, although *FLC* accounts for a large part of the high germination rate for *flk-2 hos5-5* seeds, *FLC*-independent factors contribute to a minor fraction of their germination vigor, mirroring our observations on flowering. These results reinforce the importance of the interaction between *FLK* and *HOS5* in regulating *FLC*, and extend its relevance to another fundamental aspect of plant reproduction: seed germination.

### Genome-wide profiling suggests that *FLK* and *HOS5* interact to limit the expression of stress-inducible genes

In addition to regulating *FLC*, *FLK* and *HOS5* participate in stress and defense responses (Chen *et al.*, 2013; Guan *et al.*, 2013; Jiang *et al.*, 2013; Thatcher *et al.*, 2015; Fabian *et al.*, 2023), suggesting that *FLK* and *HOS5* are likely to participate in a regulatory hub that integrates flowering and stress response pathways. To delve into the transcriptomic landscape influenced by *FLK* and *HOS5*, we performed RNA sequencing (RNA-seq) experiments. RNA was isolated from Col-0, *flk-2*, *hos5-5*, and *flk-2 hos5-5* plants grown under long-day conditions. Our RNA-seq analysis pipeline [false discovery rate (FDR) threshold of 5%] uncovered numerous differentially expressed genes (DEGs) relative to the wild type (Supplementary Table S2). We identified 762 and 433 genes less expressed in *flk-2* and *hos5-5*, respectively, including 189 common genes (Supplementary Fig. S7). We also found 590 significantly up-regulated genes in *flk-2*, and 815 in *hos5-5*, with 356 being common to both groups (Supplementary Fig. S7), indicating that *FLK* and *HOS5* share common downstream genes.

Interestingly, we detected 3348 DEGs in the *flk-2 hos5-5* double mutant, nearly three times the number found in each single mutant. Among them, 1533 loci were down-regulated whereas the other 1815 were significantly expressed above wild-type levels (Supplementary Fig. S7; Supplementary Table S2). Furthermore, the striking increase of DEGs in *flk-2 hos5-5* plants revealed a high number of genes specifically altered in the double mutant (986 down- and 1024 up-regulated loci; Supplementary Fig. S7). All these results together suggests that *FLK* and *HOS5* are broad-spectrum regulatory genes that most probably act cooperatively, as supported by their protein and genetic interactions. Transcriptomic profiling was validated by qRT–PCR expression analyses of *FLC* and additional genes, which largely mirrored RNA-seq abundance profiles (Fig. 2; Supplementary Figs S5, S8; Supplementary Table S2).

To further our understanding of the processes influenced by *FLK* and *HOS5*, we performed an enrichment analysis using the GO database. Many over-represented GO terms for biological processes were related to stress responses (Supplementary Table S3). Enriched GO terms, such as ‘cellular response to hypoxia’, ‘response to cold’, ‘defense response to fungus’, ‘defense response to bacterium’, ‘response to salicylic acid’, or ‘response to oxidative stress’, were identified from up-regulated genes for the three mutant backgrounds evaluated. The *hos5-5* and *flk-2 hos5-5* mutants also showed enrichment for the GO term ‘innate immune response’, whereas both *flk-2* and *flk-2 hos5-5* exhibited enrichment in ‘response to salt stress’ and JA-associated GO terms (Supplementary Table S3). The conspicuous enrichment in stress- and defense-related GO terms among the up-regulated *flk-2 hos5-5* DEGs strongly suggests that *FLK* and *HOS5* cooperate to restrict the expression of stress-inducible genes. The remarkably high number of DEGs specifically up-regulated in *flk-2 hos5-5* is also consistent with this view.

### Increased tolerance of *flk hos5* plants to salt and oxidative stress

**Fig. 4.**
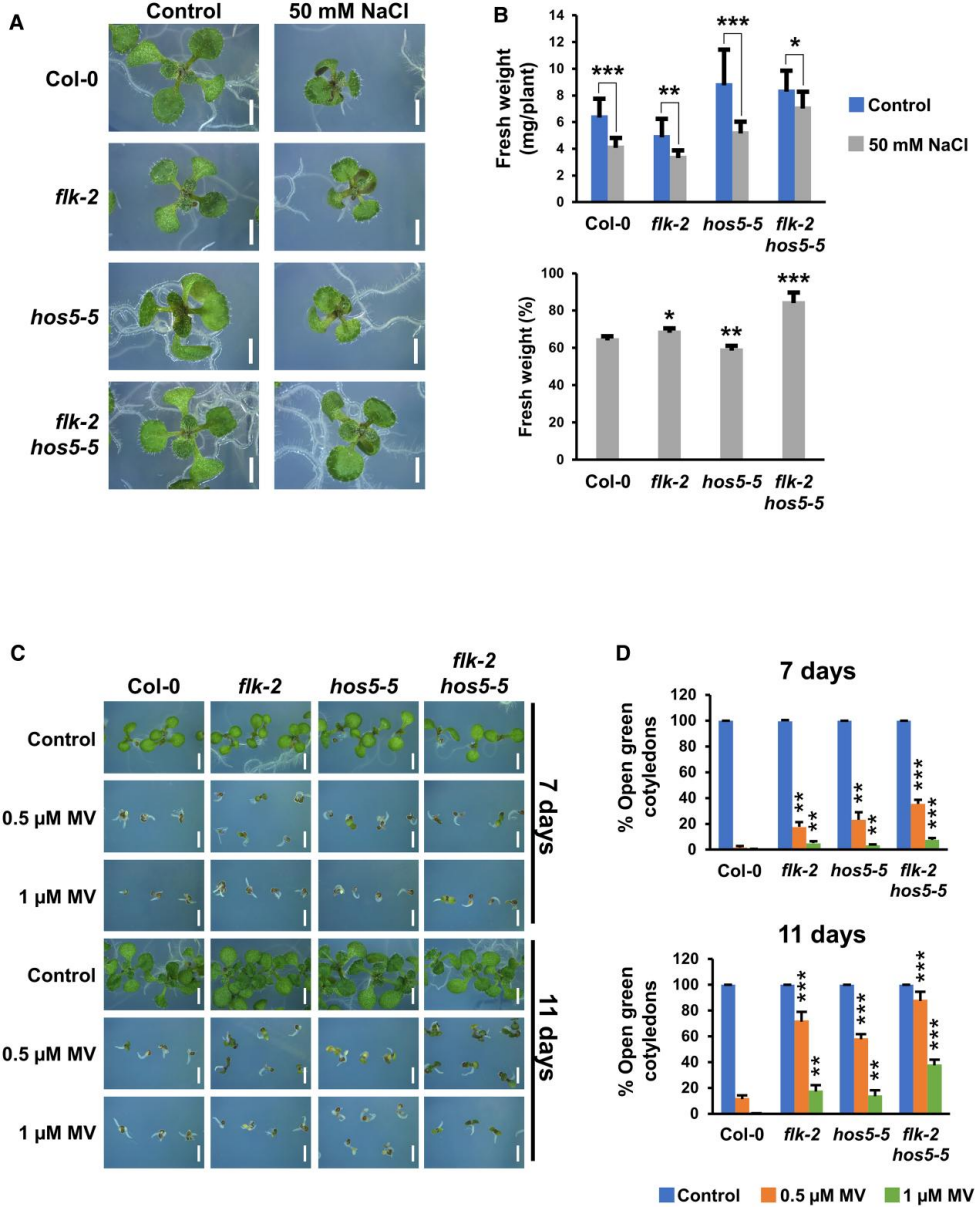
Increased tolerance of *flk hos5* plants to salt and oxidative stress. (A) Representative 10-day-old seedlings of wild-type Col-0, and mutant genotypes, grown on control medium or supplemented with 50 mM NaCl. (B) Fresh weight of 10-day-old plants grown on control medium and 50 mM NaCl. The top graph corresponds to two independent experiments with 12 plants each. Bars indicate the mean ±SD, and asterisks denote significant differences between control and NaCl-treated plants of the same genotype. The bottom graph represents the relative percentage of fresh weight of plants grown on NaCl with respect to their untreated controls. Asterisks indicate significant differences with respect to Col-0. (C) Wild-type Col-0 and mutant plants at 7 d and 11 d after stratification, grown on control medium, or supplemented with 0.5 µM or 1 µM methyl viologen (MV, Paraquat), respectively. (D) Percentage of open green cotyledons in germinated seeds at 7 d and 11 d in control medium, or in the presence of MV. Data correspond to three independent experiments with no less than 100 seedlings per genotype. Asterisks indicate significant differences with respect to untreated controls of the same genotype. *P<0.05; **P<0.01; ***P<0.001. Scale bars, 0.2 cm.

In our assays on saline medium, *flk-2 hos5-5* showed the highest germination rate. In addition, differences in post-germination development were also observed. At 150 mM NaCl, *flk-2* and *flk-2 hos5-5* double mutants showed more cotyledons and true leaves than *hos5-5* and Col-0 (Supplementary Fig. S9A). However, at 200 mM NaCl, only *flk-2 hos5-5* seedlings were still able to develop open dark-green cotyledons (Supplementary Fig. S9B), suggesting that this background might be more tolerant to salt stress conditions. We next plated seeds on 50 mM NaCl, a concentration that did not affect germination in any of the strains examined, but impacted biomass accumulation (Fig. 4A). Under these conditions, *hos5-5* seedlings appeared more affected than *flk-2* and Col-0 (Fig. 4B), consistent with *hos5* sensitivity to salt stress (Chen *et al.*, 2013). Conversely, weight loss in *flk-2 hos5-5* plants was very moderate, suggesting that double mutants adapt better to saline/osmotic stress than single mutant and wild-type individuals (Fig. 4B). These results nicely correlated with transcriptomic enrichment in *flk-2 hos5-5* of salt response genes, including, among others, *CBL-INTERACTING PROTEIN KINASE21* (*CIPK21*), *WRKY25*, *WRKY33*, or *MYB44*, whose overexpression enhances Arabidopsis salt tolerance (Jung *et al.*, 2008; Jiang and Deyholos, 2009; Pandey *et al.*, 2015; Supplementary Table S2).

Detrimental effects of salt stress, aside from osmotic and ionic imbalance, often lead to reactive oxygen species (ROS) production and subsequent oxidative damage, a secondary effect common to other types of stress (Fichman and Mittler, 2020; Mansour and Hassan, 2022). Therefore, we tested sensitivity to the oxidative stress inducer MV. Based on the percentage of established seedlings with green open cotyledons, all examined mutant backgrounds were less sensitive to MV when compared with Col-0 (Fig. 4C, D). Indeed, resistance of *flk* to oxidative stress was previously reported (Fabian *et al.*, 2023). However, although the GO term ‘response to oxidative stress’ was enriched in all three mutant backgrounds (Supplementary Table S3), *flk-2 hos5-5* plants showed the most robust resistance (Fig. 4C, D). This paralleled the increased expression of numerous antioxidant activities, including peroxidases, glutathione *S*-transferases, and catalases, some of which were specifically up-regulated in the double mutant (Supplementary Table S2). Additional up-regulated genes known to promote oxidative stress tolerance included *WRKY25*, *WRKY33*, *CIPK9*, or *PATELLIN2* (*PATL2*) (Hornbergs *et al.*, 2023; Supplementary Fig. S6; Supplementary Table S2). Stress-derived ROS production mostly depends on the NADPH oxidase RESPIRATORY BURST OXIDASE HOMOLOG D (RBOHD), which is activated by BOTRYTIS INDUCED KINASE 1 (BIK1) and negatively regulated by the recently described PHAGOCYTOSIS OXIDASE/BEM1P (PB1) DOMAIN-CONTAINING PROTEIN (PB1CP) (Goto *et al.*, 2024). Interestingly, all were found to be up-regulated in *flk-2 hos5-5* plants, probably contributing to fine-tune the final output of ROS production (Supplementary Table S2). These assays functionally validate our RNA-seq data and further support a role for *FLK* and *HOS5* in abiotic stress responses.

### The *flk hos5* double mutant exhibits augmented salicylic acid and jasmonic acid levels and increased resistance to fungal infection

The expression profiles of loci related to SA and JA biosynthesis/signaling pathways displayed clear differences between *flk-2* and *hos5-5* single mutants. For example, the expression of SA-related genes, such as *PHYTOALEXIN DEFICIENT 4* (*PAD4*), *ISOCHORISMATE SYNTHASE 1* (*ICS1*), and *ACCELERATED CELL DEATH 6* (*ACD6*) (Dempsey *et al.*, 2011), decreased in *flk-2*, whereas in *hos5-5* plants, they either increased or remained unchanged. Accordingly, the SA marker *PATHOGENESIS-RELATED GENE 1* (*PR1*) (Jung and Hwang, 2000) was down-regulated in *flk-2* but up-regulated in *hos5-5* (Supplementary Table S2). These results agree with *FLK*-positive regulation of SA-mediated defense (Fabian *et al.*, 2023).

On the other hand, JA-associated GO terms were over-represented among *hos5-5* down-regulated genes (Supplementary Table S3), as observed in the allelic mutant *esr1-1* (Thatcher *et al.*, 2015). Conversely, these terms were enriched among *flk-2* up-regulated activities, including key genes for JA biosynthesis or signaling such as *LIPOXYGENASE 2* (*LOX2*), *LOX3*, *ALLENE OXIDE SYNTHASE* (*AOS*), *ALLENE OXIDE CYCLASE 2* (*AOC2*), *MYC2*, and *VEGETATIVE STORAGE PROTEIN 1* (*VSP1*) (Wasternack and Hause, 2013; Supplementary Table S2).

Antagonistic interactions between JA and SA are well documented in Arabidopsis (Hou and Tsuda, 2022). However, *flk-2 hos5-5* double mutants seemed to recapitulate features from both single mutants. Some SA key genes were up-regulated (e.g. *PAD4*) or unchanged (e.g. *ICS1*), but still maintained high levels of *PR1* expression (Supplementary Table S2). Notably, the SA receptor-encoding gene *NONEXPRESSOR OF PR GENES 1* (*NPR1*) (Zavaliev and Dong, 2024) was significantly up-regulated only in *flk-2 hos5-5* plants (Supplementary Table S2). On the other hand, JA-related genes, including two of the most characteristic JA activity markers, *MYC2* and *PDF1.2* (Wasternack and Hause, 2013), also showed high transcript abundance in *flk-2 hos5-5*, as well as key genes for plant growth–defense trade-off under JA signaling (e.g. *MYB44*, *WRKY18*, *WRKY33*, and *ORA47*) (Zhang *et al.*, 2020a; Wang and Zhang, 2021; Supplementary Table S2).

**Fig. 5.**
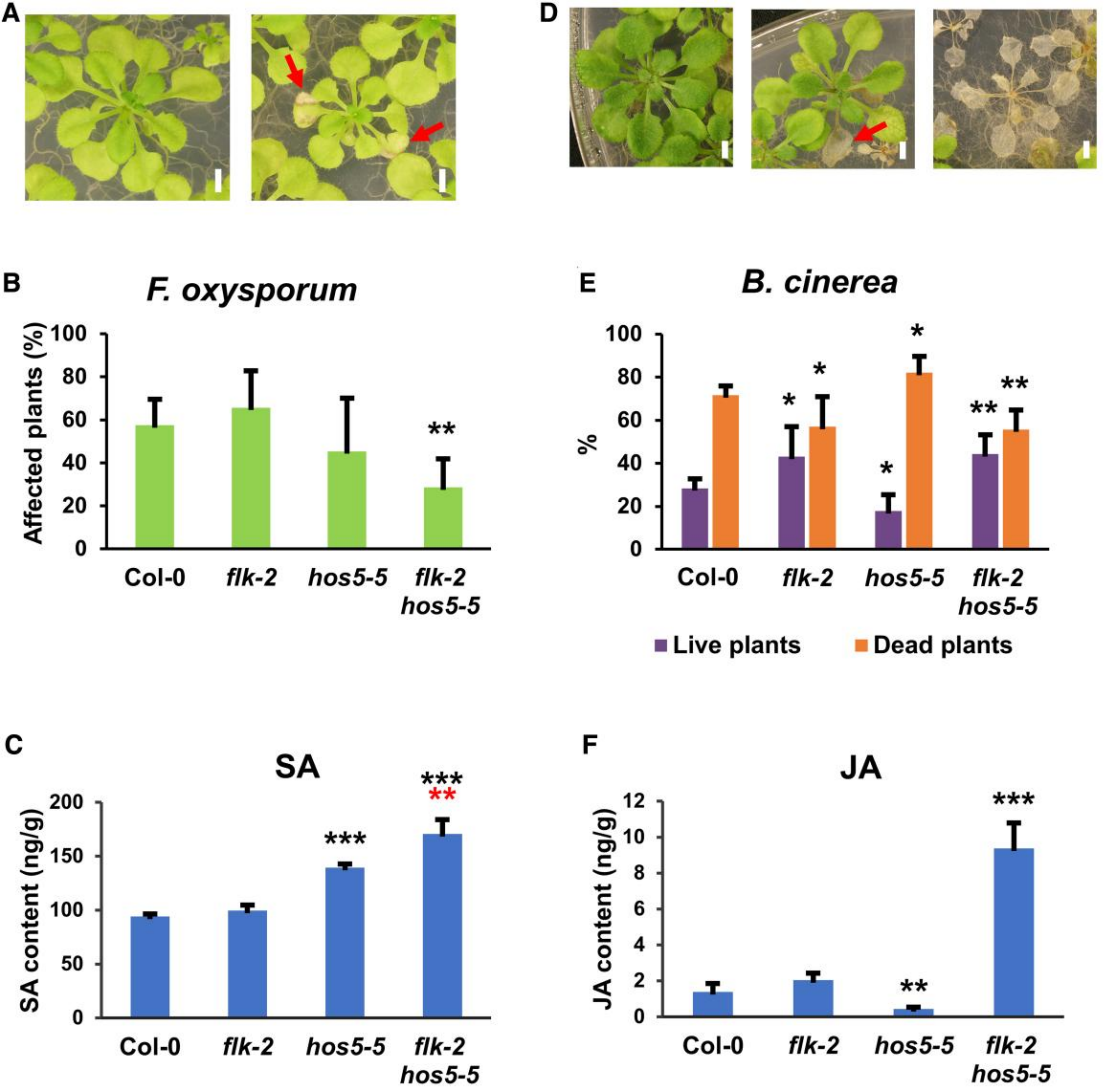
Increased resistance of the *flk hos5* mutants to fungal pathogen infection. (A) Representative asymptomatic (left) and affected (right) 2-week-old plants infected with *F. oxysporum* and photographed at 15 dai (days after infection). (B) Average percentage of plants showing *F. oxysporum* disease symptoms at 15 dai. (C) Total SA content in 12-day-old wild-type and mutant plants. (D) Representative 2-week-old plants infected with *B. cinerea* and photographed at 15 dai. Asymptomatic plants (left), live plants with necrotic lesions (middle), and dead plants (right). (E) Average percentage of live (purple) and dead (orange) plants infected with *B. cinerea* at 15 dai. (F) Total JA content in 12-day-old wild-type and mutant plants. For fungal inoculations, three biological replicates were carried out, each containing at least 40 plants per genotype. For hormone measurement, data represent the mean value of three replicates with at least 20 plants per sample. Bars indicate the mean ±SD. Black and red asterisks indicate significant differences with respect to corresponding Col-0 controls and *hos5-5* plants, respectively (*P<0.05; **P<0.01; ***P<0.001). Red arrows in (A) and (D) indicate disease lesions in live plants. Scale bars, 2mm.

SA and JA play crucial roles in plant immunity against biotrophic/hemibiotrophic and necrotrophic pathogens, respectively (Zhang *et al.*, 2020b). Molecular signatures for SA and JA activities in our transcriptomic dataset prompted us to test the susceptibility of *flk*/*hos5* mutants to phytopathogenic fungi with different lifestyles. Plants were inoculated with the hemibiotroph *F. oxysporum*, responsible for wilt disease. Col-0 and *flk-2* mutants did not show significant differences when exposed to *F. oxysporum* (Fig. 5A, B), and their endogenous SA levels were also very similar (Fig. 5C), despite lower expression of key SA-related genes in *flk-2*. By contrast, the *hos5-5* mutant exhibited higher endogenous SA levels than Col-0, although higher resistance to *F. oxysporum* was not statistically significant, probably due to interassay variability in this mutant (Fig. 5B, C). Actually, the *hos5* allele *esr1-1* was reported to be more resistant to wilt disease (Thatcher *et al.*, 2015). Interestingly, the *flk-2 hos5-5* double mutants were clearly more resistant to *F. oxysporum* (Fig. 5B), and their endogenous SA levels were significantly higher than those of *hos5-5* plants (Fig. 5C).

We also challenged our mutant strains with *B. cinerea*, a necrotrophic fungus causing gray mold disease in many plant species, including crops (Bi *et al.*, 2023). The ratio between dead and live plants indicated that *hos5-5* was more susceptible than Col-0, whereas *flk-2* and *flk-2 hos5-5* mutants were more resistant (Fig. 5D, E). Increased resistance to *B. cinerea* by *flk* nicely fits with the enrichment of JA-related GO terms in *flk-2* and *flk-2 hos5-5* (Supplementary Table S3), and it has been recently reported by another group (Fabian *et al.*, 2023). Also consistent with transcriptomic data and disease severity, JA levels in *hos5-5* were significantly lower than those of Col-0 (Fig. 5F). Remarkably, the endogenous JA level in *flk-2 hos5-5* double mutants was very high, exceeding by far that of *flk-2* plants (Fig. 5F). This was surprising because, despite such a difference in JA content, resistance to *B. cinerea* was very similar in both mutants (Fig. 5E, F).

High investment in defense is usually associated with growth or developmental trade-offs (Karasov *et al.*, 2017; Zhang *et al.*, 2020b). However, no morphological anomalies were detected in *flk-2 hos5-5*, prompting us to consider uncoupling of stress and growth limitation. We therefore decided to monitor primary root growth. Plants with high endogenous JA levels typically exhibit a short-root phenotype and sensitivity to the JA derivative MeJA (Wasternack and Hause, 2013). However, *flk-2 hos5-5* roots were similar to those of Col-0 (Supplementary Fig. S10A). Additionally, mutant and wild-type roots did not differ much when exposed to increasing MeJA concentrations. Only at 50 µM MeJA were *flk-2 hos5-5* roots slightly shorter (Supplementary Fig. S10). These results suggest that stress tolerance and growth restriction might be uncoupled, as previously postulated for *hos5* (Thatcher *et al.*, 2015). The growth-inhibitory effect of JA might be counteracted by other misregulated gene activities. For instance, the JA negative regulator *JAM1*/*bHLH17*, whose overexpression attenuates JA-mediated root inhibition (Han *et al.*, 2023), is up-regulated in the three mutant backgrounds (Supplementary Table S2). Likewise, endogenous JA levels might be modulated by negative feedback mechanisms (Gasperini and Howe, 2024). Accordingly, some genes encoding JA catabolic enzymes, such as *SULFOTRANSFERASE 2A* (*ST2A*), which is enhanced by JA treatments (Gidda *et al.*, 2003), were also up-regulated in *flk-2 hos5-5* (Supplementary Table S2). We also considered the possibility that, despite a large difference in JA content, the levels of the canonical bioactive hormone jasmonoyl-isoleucine (JA-Ile) (Fonseca *et al.*, 2009; Wasternack and Hause, 2013) could explain the similar response to *B. cinerea* by *flk* and *flk hos5*. Therefore, we measured specifically the levels of JA-Ile in our mutants. In line with JA abundance, however, we observed a very similar fold increase in *flk-2 hos5-5* JA-Ile content when compared with *flk-2* plants (Supplementary Fig. S11).

### mRNA regulation mediated by *FLK* and *HOS5*

**Fig. 6.**
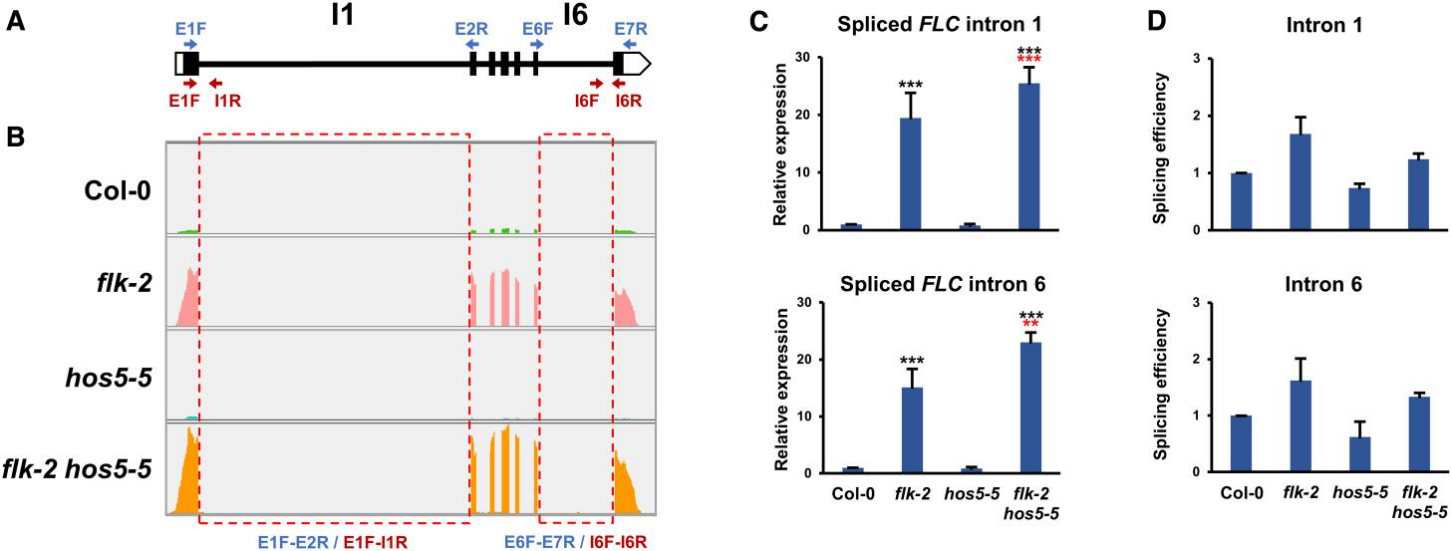
Splicing efficiency of *FLC* pre-mRNA in the *flk-2*, *hos5-5*, and *flk-2 hos5-5* mutants. (A) Schematic representation of the *FLC* gene (isoform 1). Thick bars indicate exons (black, translated; white, untranslated). Thin lines denote introns. Blue and red arrowheads indicate positions of primers used to amplify spliced and unspliced products, respectively. E1F (Exon 1 Forward); E2R (Exon 2 Reverse); E6F (Exon 6 Forward); E7R (Exon 7 Reverse); I1R (Intron 1 Reverse); I5F (Intron 6 Forward); I6R (Intron 6 reverse). The numbers of examined introns are indicated on top. (B) Wiggle plots of *FLC* RNA-seq data in Col-0 and the indicated mutant backgrounds. Read counts were normalized as determined by IGV software. The dashed boxes indicate introns analyzed in (C) and (D). (C) Relative expression of *FLC* monitored by qRT–PCR as the amount of spliced intron 1 (top) or spliced intron 6 (bottom) transcripts. (D) Splicing efficiency (measured as the ratio of the accumulation of spliced forms to unspliced forms) of introns 1 and 6, respectively, as indicated at the bottom of (B). For (C) and (D), data correspond to three biological replicates with three technical replicates each. Bars represent the mean ±SD. Black and red asterisks indicate significant differences with respect to Col-0 and *flk-2* plants, respectively (**P<0.01; ***P<0.001).

Our results corroborate that *FLK* and *HOS5* act in concert to control floral transition through *FLC* regulation, and that they also orchestrate stress responses by regulating additional genes, including stress-related loci (see above). To gather information on how *flk* and *hos5* mutations jointly impact mRNA expression, we first explored *FLC* regulation*. FLK* has been reported to affect *FLC* splicing efficiency (Amara *et al.*, 2023). We therefore monitored spliced and unspliced *FLC* transcripts corresponding to the large intron 1, common to all *FLC* isoforms, and the terminal intron 6 of *FLC* variant 1 (Fig. 6A). In *flk-2*, the levels of spliced products increased significantly more than those of their respective unspliced forms (Fig. 6B, C), with splicing efficiency (ratio of spliced over unspliced transcripts) being higher than in Col-0 (Fig. 6D). This aligns with recent findings indicating that FLK binds to *FLC* mRNA in an m^6^A-dependent manner to impair splicing (Amara *et al.*, 2023). On the other hand, levels of correctly spliced forms in the *hos5-5* mutants were slightly lower than those in Col-0 which, together with a modest increment of unspliced forms, led to reduced splicing efficiency as a result (Fig. 6B–D). In fact, splicing efficiency of both introns in *flk-2 hos5-5*, although higher than in Col-0, was lower than that of *flk-2*, despite the notable increase of spliced products (Fig. 6B–D). *HOS5* affects splicing but also impairs transcription by preventing 5′ capping (Chen *et al.*, 2013; Jiang *et al.*, 2013). Therefore, and as previously reported for *flk*, high *FLC* abundance may result from increased splicing efficiency (Amara *et al.*, 2023). Nevertheless, enhanced transcription and/or increased transcript stability cannot be ruled out in *flk-2 hos5-5* plants.

**Fig. 7.**
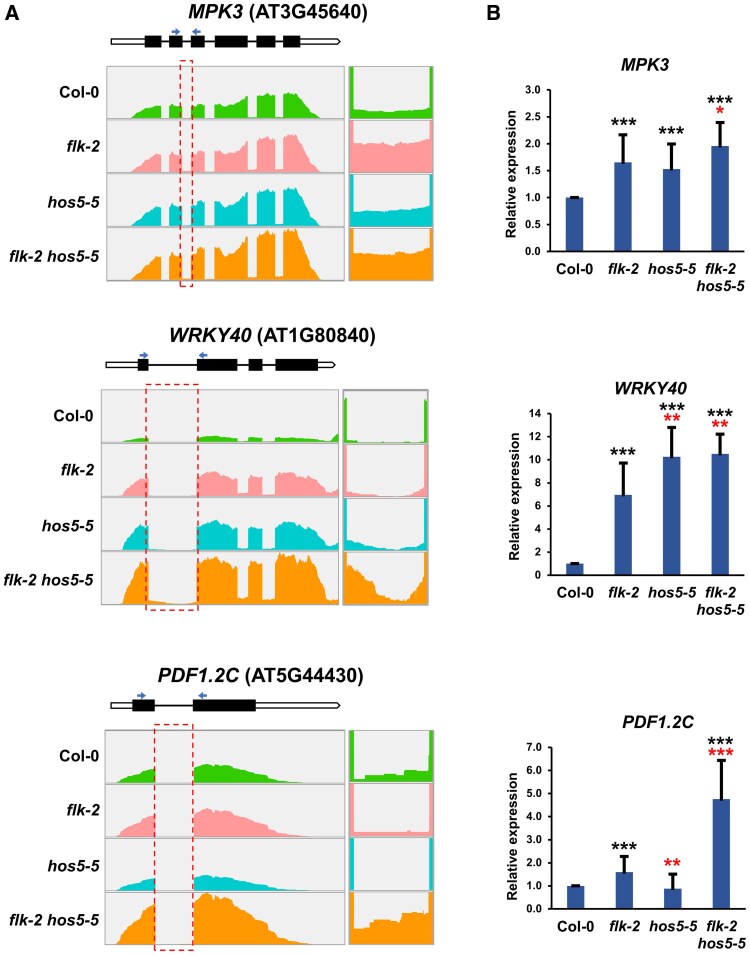
Up-regulated intron sequences in up-regulated genes. (A) Top of each panel: schematic representation of the corresponding gene. Thick bars indicate exons (black, translated; white, untranslated). Thin lines denote introns. Arrowheads indicate the positions of primers used to amplify the corresponding spliced PCR products. Bottom: wiggle plots of RNA-seq data in Col-0 and mutant backgrounds. Read counts were normalized as determined by IGV software. The dashed boxes indicate introns analyzed in the right panels, a magnification of which is shown on the right. In these examples, the indicated intron-specific reads parallel the mRNA expression level in each genotype (see also Supplementary Fig. S13). (B) Relative gene expression monitored by qRT–PCR as the amount of the indicated spliced transcripts. Data correspond to three biological replicates with three technical replicates each. Bars represent the mean ±SD. Black and red asterisks indicate significant differences with respect to Col-0 and *flk-2* plants, respectively (*P<0.05; **P<0.01; ***P<0.001).

To gain a broader perspective, we further searched our RNA-seq datasets for introns differentially expressed in our mutants relative to the wild type. In *flk-2*, we found 228 differentially up-regulated introns (intron-specific reads more abundant than in Col-0), corresponding to 97 genes, 37 of which were up-regulated in this mutant, whereas only one was down-regulated (Fig. 7; Supplementary Figs S12A, S13; Supplementary Table S4). Likewise, 119 introns, representing 47 genes, were up-regulated in *hos5-5*. Among the genes involved, 33 were up-regulated in *hos5-5*, and none was down-regulated (Fig. 7; Supplementary Figs S12A, S13; Supplementary Table S4). Remarkably, we found 1328 up-regulated introns in the *flk-2 hos5-5* double mutant, significantly more than in either single mutant. This set represented 450 genes, 301 of which were up-regulated in *flk-2 hos5-5*, most of them being double mutant specific (253, 84%) (Fig. 7; Supplementary Figs S12A, S13; Supplementary Table S4). By contrast, we detected only nine down-regulated genes in this group, including that found among *flk-2* down-regulated genes (Supplementary Fig. S12A).

The above data suggest that intron retention, a frequent outcome of splicing alteration in plants (Reddy *et al.*, 2012), is not a prominent mechanism of gene repression in our mutant backgrounds. This agrees with previous results indicating that no significant intron retention takes place in the *hos5* mutant under normal (non-stress) conditions (Chen *et al.*, 2013). In line with this notion, no up-regulated genes harboring down-regulated introns (fewer intron-specific reads than in Col-0) were found in any of the three mutant genotypes (Supplementary Figs S12B, S14; Supplementary Table S4). These data indicate a positive correlation between DEGs and their differentially expressed introns. With very few exceptions, up-regulated introns appeared in up-regulated DEGs, whereas all down-regulated introns were found in down-regulated DEGs (Supplementary Fig. S12). Taken together, our results may be consistent with FLK and HOS5 actions on mRNA maturation, including splicing, since higher splicing efficiency of featured introns in the *flk-2* mutant was observed in some up-regulated genes (Supplementary Figs S8, S15), but may also reflect additional effects on transcript stability and/or transcription rate, particularly in *flk-2 hos5-5* plants.

## Discussion

Plants have evolved the ability to adjust their flowering time to environmental fluctuations and stress conditions (Kazan and Lyons, 2016). Regulatory genes involved in floral timing and stress are crucial, probably serving as key molecular hubs to adequately integrate these responses. Here, we provide evidence based on genetic, molecular, physiological, and transcriptomic analyses that delineates the KH-domain genes *FLK* and *HOS5* as an integrating regulatory node that couples flowering and stress responses to secure plant survival and reproductive success.

### *HOS5* cooperates with *FLK* to repress *FLC* and promote flowering

We have demonstrated that *HOS5* strongly interacts with *FLK* to promote the floral transition via *FLC* repression. The role of *HOS5* as a floral regulator has been previously overlooked due to the weak effect of *hos5* mutant alleles on flowering. In contrast, *hos5* led to a dramatic increase of *FLC* levels and flowering time when combined with *flk*. This effect was observed in both long- and short-day regimes, being more pronounced in the latter case (Supplementary Fig. S4), possibly due to higher expression of flowering repressors under this light regime (Wang *et al.*, 2025). Supporting its role in flowering, *HOS5* shows expression peaks in the vegetative and reproductive apices (Karlsson *et al.*, 2015). Moreover, the contribution of *hos5* to *FLC* overexpression was also evidenced by the enhanced *FLC*-dependent germination rates of *flk-2 hos5* seeds under salt stress.

### *FLK* and *HOS5* jointly modulate the expression of stress-inducible genes

Aside from *FLC*, our RNA-seq data analysis revealed a substantial overlap of DEGs between the *flk-2* and *hos5-5* single mutants. Additionally, DEGs found in *flk-2 hos5-5* were about three times those in either single mutant, most of them specific to the double mutant. This interesting feature, together with the enrichment in stress-related GO terms, suggests that, besides their independent functions, co-regulation via *FLK–HOS5* plays an important role in the modulation of stress-related genes, a scenario reinforced by the association of both proteins *in planta*. It is tempting to anticipate that a fraction of them could be identified as direct targets. However, additional work beyond the scope of this study is required to verify this.

The prevalence of stress-related GO categories in *flk-2* and *hos5-5* agreed with previous reports for allelic mutations (Thatcher *et al.*, 2015; Fabian *et al.*, 2023). Remarkably, further enrichment of stress-related functions was observed among DEGs in *flk-2 hos5-5*, including terms related to salt and oxidative stress responses. In line with this, the *flk-2 hos5-5* mutant thrived better on saline medium, and showed less sensitivity to oxidative stress than Col-0 and single mutants. Numerous gene activities that confer salt tolerance and/or alleviate oxidative damage were specifically up-regulated in *flk-2 hos5-5*, when compared with single mutants. This could probably mitigate ROS-dependent deleterious effects and improve tolerance to salt stress.

Our results also suggest that, together, *FLK* and *HOS5* regulate responses to biotic agents and defense hormone homeostasis. The resistance of *hos5* to *F. oxysporum* and that of *flk* against *B. cinerea* agreed with previous studies (Thatcher *et al.*, 2015; Fabian *et al.*, 2023). Conversely, *hos5-5* was more susceptible to *B. cinerea*, which aligns with the reduced expression of JA-related genes and lower JA content (Fig. 5). Remarkably, *flk-2 hos5-5* doubles additively combined characteristics of each single mutant: enhanced resistance to both fungal pathogens and higher levels of JA and SA. Both hormones usually act antagonistically. However, cooperative and synergistic effects have also been observed in diverse species, including Arabidopsis, indicating coordinated activation of JA/SA signaling when required (Mur *et al.*, 2006; Zhang *et al.*, 2020c; Hou and Tsuda, 2022). Simultaneous deficiency of *FLK* and *HOS5* might mimic this scenario. In fact, synergistic effects of SA and JA on the expression of their respective markers *PR1* and *PDF1.2* are documented (Zhang *et al.*, 2020c), and both genes are up-regulated in *flk-2 hos5-5* (Supplementary Table S2). Consistently, numerous genes involved in SA and JA responses were highly up-regulated in the double mutant, potentially contributing to fungal resistance, including the key general stress regulators *ORA47* (Zeng *et al.*, 2022) and *WRKY33*. The latter is a crucial gene for defense against necrotrophic fungi (Zheng *et al.*, 2006), which also collaborates with the SA master regulator *NPR1* to mediate systemic acquired resistance (SAR) (Li *et al.*, 2018; Wang *et al.*, 2018). Recent analyses also suggest that some JA-responsive genes could enhance SA-mediated immunity, such as *MYB44*, which is up-regulated in *flk-2* and yet is significantly more abundant in *flk-2 hos5-5* (Zhang *et al.*, 2020c; Zeng *et al.*, 2022; Supplementary Table S2).

### Stress tolerance and moderate fitness cost in *flk hos5* plants

Up-regulation of ‘stress genes’ and elevated SA and JA levels leads to increased tolerance of the *flk-2 hos5-5* double mutant to biotic and abiotic stress. However, no signs of growth limitation were observed in such plants. Uncoupled stress tolerance and growth was previously proposed for *hos5* mutants (Thatcher *et al.*, 2015), and no evidence of impaired growth has been described for *flk* or when combined with other AP mutants (Veley and Michaels, 2008). In *flk-2 hos5-5* mutants, SA and JA activities might be regulated mainly at the signaling/perception level, perhaps favoring particular hormone branches that allow tolerance without yield cost. Alternatively, but not mutually exclusively, misregulated counteracting activities might contribute to modulate their effects. In the case of JA, differential production of the bioactive conjugate JA-Ile does not seem likely since JA-Ile levels are also higher in *flk-2 hos5-5*, and the genes for the conjugating enzymes, *JASMONATE RESISTANT 1* (*JAR1*) and *GH3.10* (Ni *et al.*, 2025), do not vary in our RNA-seq datasets (Supplementary Table S2). However, another possibility is the attenuation of JA signaling through JA-Ile turnover. Expression of *CYP94C1* (Aubert *et al.*, 2015), as well as that of the amido-hydrolase-encoding gene *IAA-LEUCINE RESISTANT (ILR)-LIKE GENE 6* (*ILL6*; Widemann *et al.*, 2013), increases significantly in *flk-2 hos5-5* in comparison with *flk*-2, which might reduce JA signaling in the double mutant. This is consistent with increased expression of *MYB47* in *flk-2 hos5-5* (Supplementary Table S2). JA induces *MYB47* which, in turn, positively regulates *CYP94C1* in a negative feedback loop that fine-tunes JA signaling (Cao *et al*., 2025). Consistent with this, root growth in Col-0 and mutants did not differ during MeJA inhibition assays (Supplementary Fig. S10), as already reported for *hos5* (Thatcher *et al.*, 2015).

### mRNA expression regulatory mechanisms mediated by *FLK* and *HOS5*

In agreement with recent results (Amara *et al.*, 2023), splicing efficiency of *FLC* introns increased in our *flk-2* mutants. Notably, *FLC* splicing efficiency in *flk-2 hos5-5* was lower than that in *flk-2*, in spite of much higher expression levels. In *hos5-5*, *FLC* splicing efficiency decreased, agreeing with HOS5 splicing regulation and its interaction with SR splicing factors (Chen *et al.*, 2013). However, HOS5 also down-regulates transcription by interfering with 5′ capping, which is required for efficient transcript elongation (Jiang *et al.*, 2013). Interestingly, other *FLC* regulators, such as the RNA recognition motif proteins RZ-1B and RZ-1C, also promote efficient splicing and repress transcription (Wu *et al.*, 2016). *FLC* overexpression in *flk-2 hos5-5* mutants may reflect dual effects on transcription and co-transcriptional processing. In the *flk-2* single mutant, increased *FLC* expression probably leads to greater transcript stability and splicing efficiency. However, further activation of transcription in *flk-2 hos5-5* should result from efficient 5′ capping after loss of *HOS5* activity. A similar mode of action is seen for FRIGIDA (FRI), which up-regulates *FLC* co-transcriptionally by direct physical interaction with the nuclear cap-binding complex (Geraldo *et al.*, 2009). In budding yeast, slowing the RNA polymerase II elongation increases splicing efficiency, whereas faster elongation reduces it (Aslanzadeh *et al.*, 2018). This negative correlation might be adopted to explain why *FLC* (and some other genes under the influence of *FLK*/*HOS5*) further increases its expression levels in *flk-2 hos5-5* with respect to *flk-2* despite lower intron splicing efficiency.

*FLK* and *HOS5* might also cooperate in additional RNA regulatory mechanisms. The lack of *HOS5* has been previously linked to altered polyadenylation site selection in some stress-inducible genes (Jiang *et al.*, 2013), while FLK participates in *AG* transcript termination (Rodríguez-Cazorla *et al.*, 2015), consistent with a possible role for *FLK*/*HOS5* in regulating RNA 3′-end formation. This, in turn, might also impact on transcript stability, as demonstrated by the negative role of *FLK* on *FLC* (Amara *et al.*, 2023).

On the other hand, our analysis of the RNA-seq datasets suggests a minor role for splicing in explaining the global differential gene expression seen in the mutant backgrounds screened here, with very few exceptions. We observed a positive correlation between relative levels of DEGs and those of their differentially expressed introns. Thus, intron retention does not seem to be a prevailing mechanism to limit gene expression in *flk/hos5* backgrounds, at least under normal growth conditions. This was previously observed for *hos5*, in which intron retention was only detected when plants were grown on saline medium (Chen *et al.*, 2013).

**Fig. 8.**
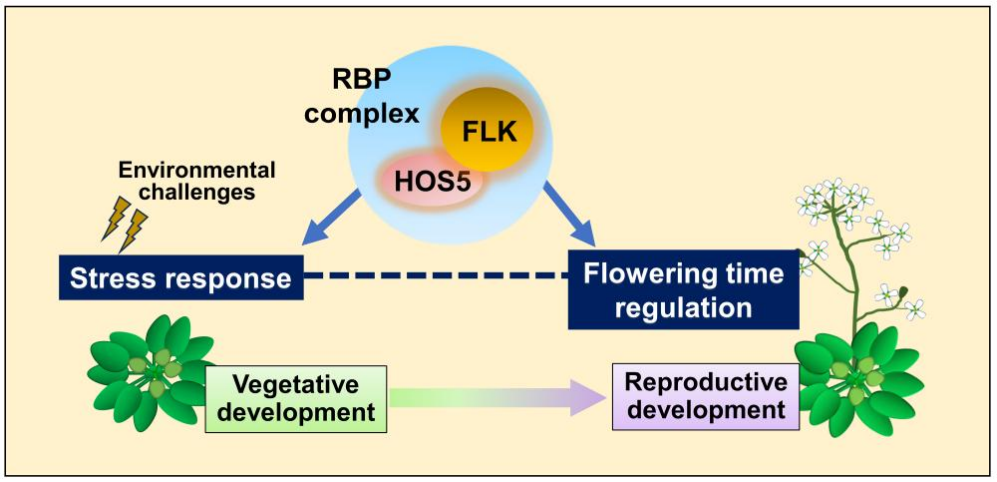
A working model in which *FLK*, a key flowering time regulator, and *HOS5*, involved in stress responses, encode two Arabidopsis RNA-binding proteins (RBPs) with K-homology (KH) domains that interact to coordinate gene expression. Their multifaceted roles may enable rapid co-transcriptional regulation, necessary to integrate flowering and stress response pathways to adapt plant responses to endogenous and environmental changes, thus enhancing survival and reproduction.

In summary, and considering the available data and our results, we propose that *FLK* and *HOS5* are part of a gene regulatory module for coordinating flowering (via *FLC* repression) and biotic/abiotic stress responses, which is probably recruited to adapt developmental responses, including floral transition, reproduction, and germination, to unfavorable environmental conditions (Fig. 8). Further dissection of the underlying regulatory mechanisms controlled by *FLK–HOS5* in modulating growth–defense status may provide valuable insights for translational strategies aimed at generating stress-tolerant crop varieties without developmental constraints.

## Supplementary Material

eraf286_Supplementary_Data

## Data Availability

All data supporting the findings of this study are available within the paper and within its supplementary data published online. The RNA-seq data underlying this article are available in EMBL-EBI (EMBL’s European Bioinformatics Institute) at https://www.ebi.ac.uk/biostudies/arrayexpress/studies/E-MTAB-14644, and can be accessed with accession number E-MTAB-14644.

## Supplementary data

The following supplementary data are available at *JXB*  online.

Fig. S1. Control assays for Y2H and BiFC experiments.

Fig. S2. *hos5* mutants used in this study.

Fig. S3. Vegetative rosettes of mutant and wild-type plants.

Fig. S4. Flowering time in *flk-2 hos5-5* under short-day conditions.

Fig. S5. Transcript levels of *AP1*, *SOC1*, and *TSF* in *flk–hos5* genetic backgrounds.

Fig. S6. Transcript levels of *MAF4* and *MAF5* genes in the *flk hos5 flc* genetic background.

Fig. S7. Venn diagrams for differentially expressed genes in the mutants under analysis.

Fig. S8. qRT–PCR validation of the transcriptomic RNA-seq datasets.

Fig. S9. Post-germinative development of *flk–hos5* mutant combinations under salt stress.

Fig. S10. MeJA root growth inhibition assays.

Fig. S11. JA-Ile content in *flk-2* and *flk-2 hos5-5* mutant plants.

Fig. S12. Venn diagrams for differentially expressed introns and genes in the mutants under analysis.

Fig. S13. Up-regulated intron sequences in *flk*, *hos5*, and *flk hos5* up-regulated genes.

Fig. S14. Down-regulated intron sequences in selected *flk*, *hos5*, and *flk hos5* down-regulated genes.

Fig. S15. Splicing efficiency of selected genes in the *flk*/*hos5* mutants.

Table S1. Information on molecular genotyping, qRT–PCR, and oligonucleotides, and supplementary references.

Table S2. Differentially expressed genes in *flk*, *hos5*, and *flk hos5*.

Table S3. Over-represented GO terms. Biological process.

Table S4. Differentially expressed introns in *flk*, *hos5*, and *flk hos5*.

## References

[eraf286-B1] Amara U, Hu J, Cai J, Kang H. 2023. FLK is an mRNA m6A reader that regulates floral transition by modulating the stability and splicing of *FLC* in Arabidopsis. Molecular Plant 16, 919–929. 37050878 10.1016/j.molp.2023.04.005

[eraf286-B2] Ambrosone A, Costa A, Leone A, Grillo S. 2012. Beyond transcription: RNA-binding proteins as emerging regulators of plant response to environmental constraints. Plant Science 182, 12–18. 22118611 10.1016/j.plantsci.2011.02.004

[eraf286-B3] Anders S, Pyl PT, Huber W. 2015. HTSeq—a python framework to work with high-throughput sequencing data. Bioinformatics 31, 166–169. 25260700 10.1093/bioinformatics/btu638 PMC4287950

[eraf286-B4] Aslanzadeh V, Huang Y, Sanguinetti G, Beggs JD. 2018. Transcription rate strongly affects splicing fidelity and cotranscriptionality in budding yeast. Genome Research 28, 203–213. 29254943 10.1101/gr.225615.117 PMC5793784

[eraf286-B5] Aubert Y, Widemann E, Miesch L, Pinot F, Heitz T. 2015. CYP94-mediated jasmonoyl-isoleucine hormone oxidation shapes jasmonate profiles and attenuates defence responses to *Botrytis cinerea* infection. Journal of Experimental Botany 66, 3879–3892. 25903915 10.1093/jxb/erv190 PMC4473988

[eraf286-B6] Bentley DL . 2014. Coupling mRNA processing with transcription in time and space. Nature Reviews. Genetics 15, 163–175. 10.1038/nrg3662 PMC4304646 24514444

[eraf286-B7] Bi K, Liang Y, Mengiste T, Sharon A. 2023. Killing softly: a roadmap of *Botrytis cinerea* pathogenicity. Trends in Plant Science 28, 211–222. 36184487 10.1016/j.tplants.2022.08.024

[eraf286-B8] Bolger AM, Lohse M, Usadel B. 2014. Trimmomatic: a flexible trimmer for Illumina sequence data. Bioinformatics 30, 2114–2120. 24695404 10.1093/bioinformatics/btu170 PMC4103590

[eraf286-B9] Cai J, Hu J, Amara U, Park SJ, Li Y, Jeong D, Lee I, Xu T, Kang H. 2023. Arabidopsis N6-methyladenosine methyltransferase FIONA1 regulates floral transition by affecting the splicing of *FLC* and the stability of floral activators *SPL3* and *SEP3*. Journal of Experimental Botany 74, 864–877. 36416766 10.1093/jxb/erac461

[eraf286-B10] Cao J, Yang Q, Zhao Y, Tan S, Li S, Cheng D, Zhang R, Zhang M, Li Z. 2025. MYB47 delays leaf senescence by modulating jasmonate pathway via direct regulation of CYP94B3/CYP94C1 expression in *Arabidopsis*. New Phytologist 246, 2192–2206. 40186431 10.1111/nph.70133

[eraf286-B11] Chen T, Cui P, Chen H, Ali S, Zhang S, Xiong L. 2013. A KH-domain RNA-binding protein interacts with FIERY2/CTD phosphatase-like 1 and splicing factors and is important for pre-mRNA splicing in Arabidopsis. PLoS Genetics 9, e1003875. 24146632 10.1371/journal.pgen.1003875 PMC3798263

[eraf286-B12] Cheng Y, Kato N, Wang W, Li J, Chen X. 2003. Two RNA binding proteins, HEN4 and HUA1, act in the processing of *AGAMOUS* pre-mRNA in *Arabidopsis thaliana*. Developmental Cell 4, 53–66. 12530963 10.1016/s1534-5807(02)00399-4 PMC5135010

[eraf286-B13] Chiang GCK, Barua D, Kramera EM, Amasino RM, Donohue K. 2009. Major flowering time gene, *FLOWERING LOCUS C*, regulates seed germination in *Arabidopsis thaliana*. Proceedings of the National Academy of Sciences, USA 106, 11661–11666. 10.1073/pnas.0901367106 PMC2710639 19564609

[eraf286-B14] Dempsey DA, Vlot AC, Wildermuth MC, Klessig DF. 2011. Salicylic acid biosynthesis and metabolism. The Arabidopsis Book 9, e0156. 22303280 10.1199/tab.0156 PMC3268552

[eraf286-B15] Fabian M, Gao M, Zhang X-N, Shi J, Vrydagh L, Kim S-H, Patel P, Hu AR, Lu H. 2023. The flowering time regulator FLK controls pathogen defense in *Arabidopsis thaliana*. Plant Physiology 191, 2461–2474. 36662556 10.1093/plphys/kiad021 PMC10069895

[eraf286-B16] Fichman Y, Mittler R. 2020. Rapid systemic signaling during abiotic and biotic stresses: is the ROS wave master of all trades? Plant Journal 102, 887–896. 10.1111/tpj.14685 31943489

[eraf286-B17] Fonseca S, Chini A, Hamberg M, Adie B, Porzel A, Kramell R, Miersch O, Wasternack C, Solano RF. 2009. (+)-7-iso-Jasmonoyl-L-isoleucine is the endogenous bioactive jasmonate. Nature Chemical Biology 5, 344–350. 19349968 10.1038/nchembio.161

[eraf286-B18] Freytes SN, Canelo M, Cerdán PD. 2021. Regulation of flowering time: when and where? Current Opinion in Plant Biology 63, 102049. 33975153 10.1016/j.pbi.2021.102049

[eraf286-B19] Gasperini D, Howe GA. 2024. Phytohormones in a universe of regulatory metabolites: lessons from jasmonate. Plant Physiology 195, 135–154. 38290050 10.1093/plphys/kiae045 PMC11060663

[eraf286-B20] Geraldo N, Bäurle I, Kidou SI, Hu X, Dean C. 2009. FRIGIDA delays flowering in Arabidopsis via a cotranscriptional mechanism involving direct interaction with the nuclear cap-binding complex. Plant Physiology 150, 1611–1618. 19429606 10.1104/pp.109.137448 PMC2705036

[eraf286-B21] Gibson DG . 2011. Enzymatic assembly of overlapping DNA fragments. Methods in Enzymology 498, 349–361. 21601685 10.1016/B978-0-12-385120-8.00015-2 PMC7149801

[eraf286-B22] Gidda SK, Miersch O, Levitin A, Schmidt J, Wasternack C, Varin L. 2003. Biochemical and molecular characterization of a hydroxyjasmonate sulfotransferase from *Arabidopsis thaliana*. Journal of Biological Chemistry 278, 17895–17900. 12637544 10.1074/jbc.M211943200

[eraf286-B23] Goto Y, Maki N, Sklenar J, Derbyshire P, Menke FLH, Zipfel C, Kadota Y, Shirasu K. 2024. The phagocytosis oxidase/Bem1p domain-containing protein PB1CP negatively regulates the NADPH oxidase RBOHD in plant immunity. New Phytologist 241, 1763–1779. 37823353 10.1111/nph.19302

[eraf286-B24] Guan P, Ripoll JJ, Wang R, Vuong L, Bailey-Steinitz LJ, Ye D, Crawford NM. 2017. Interacting TCP and NLP transcription factors control plant responses to nitrate availability. Proceedings of the National Academy of Sciences, USA 114, 2419–2424. 10.1073/pnas.1615676114 PMC5338533 28202720

[eraf286-B25] Guan Q, Wen C, Zeng H, Zhu J. 2013. A KH domain-containing putative RNA-binding protein is critical for heat stress-responsive gene regulation and thermotolerance in Arabidopsis. Molecular Plant 6, 386–395. 23087326 10.1093/mp/sss119

[eraf286-B26] Han X, Kui M, He K, Yang M, Du J, Jiang Y, Hu Y. 2023. Jasmonate-regulated root growth inhibition and root hair elongation. Journal of Experimental Botany 74, 1176–1185. 36346644 10.1093/jxb/erac441 PMC9923215

[eraf286-B27] Hasan MK, Brady LJ. 2024. Nucleic acid-binding KH domain proteins influence a spectrum of biological pathways including as part of membrane-localized complexes. Journal of Structural Biology: X 10, 100106. 39040530 10.1016/j.yjsbx.2024.100106 PMC11261784

[eraf286-B28] Hellens RP, Edwards EA, Leyland NR, Bean S, Mullineaux PM. 2000. Pgreen: a versatile and flexible binary Ti vector for *Agrobacterium*-mediated plant transformation. Plant Molecular Biology 42, 819–832. 10890530 10.1023/a:1006496308160

[eraf286-B29] Hornbergs J, Montag K, Loschwitz J, et al 2023. SEC14-GOLD protein PATELLIN2 binds IRON-REGULATED TRANSPORTER1 linking root iron uptake to vitamin E. Plant Physiology 192, 504–526. 36493393 10.1093/plphys/kiac563 PMC10152663

[eraf286-B30] Hou S, Tsuda K. 2022. Salicylic acid and jasmonic acid crosstalk in plant immunity. Essays in Biochemistry 66, 647–656. 35698792 10.1042/EBC20210090

[eraf286-B31] Jang S, Torti S, Coupland G. 2009. Genetic and spatial interactions between *FT*, *TSF* and *SVP* during the early stages of floral induction in Arabidopsis. The Plant Journal 60, 614–625. 19656342 10.1111/j.1365-313X.2009.03986.x

[eraf286-B32] Jeong IS, Fukudome A, Aksoy E, et al 2013. Regulation of abiotic stress signalling by Arabidopsis C-terminal domain phosphatase-like 1 requires interaction with a K-homology domain-containing protein. PLoS One 8, e80509. 24303021 10.1371/journal.pone.0080509 PMC3841200

[eraf286-B33] Jiang J, Wang B, Shen Y, Wang H, Feng Q, Shi H. 2013. The Arabidopsis RNA binding protein with K homology motifs, SHINY1, interacts with the C-terminal domain phosphatase-like 1 (CPL1) to repress stress-inducible gene expression. PLoS Genetics 9, e1003625. 23874224 10.1371/journal.pgen.1003625 PMC3708844

[eraf286-B34] Jiang Y, Deyholos MK. 2009. Functional characterization of Arabidopsis NaCl-inducible WRKY25 and WRKY33 transcription factors in abiotic stresses. Plant Molecular Biology 69, 91–105. 18839316 10.1007/s11103-008-9408-3

[eraf286-B35] Jung C, Seo JS, Han SW, Koo J, Kim CH, Song SI, Nahm H, Do CY, Cheong J-J. 2008. Overexpression of *AtMYB44* enhances stomatal closure to confer abiotic stress tolerance in transgenic Arabidopsis. Plant Physiology 146, 623–635. 18162593 10.1104/pp.107.110981 PMC2245844

[eraf286-B36] Jung HW, Hwang BK. 2000. Isolation, partial sequencing, and expression of pathogenesis-related cDNA genes from pepper leaves infected by *Xanthomonas campestris* pv. *vesicatoria*. Molecular Plant-Microbe Interactions 13, 136–142. 10656596 10.1094/MPMI.2000.13.1.136

[eraf286-B37] Jung JH, Park JH, Lee S, To TK, Kim JM, Seki M, Park CM. 2013. The cold signaling attenuator HIGH EXPRESSION OF OSMOTICALLY RESPONSIVE GENE1 activates *FLOWERING LOCUS C* transcription via chromatin remodeling under short-term cold stress in Arabidopsis. The Plant Cell 25, 4378–4390. 24220632 10.1105/tpc.113.118364 PMC3875724

[eraf286-B38] Karasov TL, Chae E, Herman JJ, Bergelson J. 2017. Mechanisms to mitigate the trade-off between growth and defense. The Plant cell 29, 666–680. 28320784 10.1105/tpc.16.00931 PMC5435432

[eraf286-B39] Karlsson P, Christie MD, Seymour DK, Wang H, Wang X, Hagmann J, Kulcheski F, Manavella PA, Poethig RS. 2015. KH domain protein RCF3 is a tissue-biased regulator of the plant miRNA biogenesis cofactor HYL1. Proceedings of the National Academy of Sciences, USA 112, 14096–14101. 10.1073/pnas.1512865112 PMC4653147 26512101

[eraf286-B40] Kazan K, Lyons R. 2016. The link between flowering time and stress tolerance. Journal of Experimental Botany 67, 47–60. 26428061 10.1093/jxb/erv441

[eraf286-B41] Kim D, Paggi JM, Park C, Bennett C, Salzberg SL. 2019. Graph-based genome alignment and genotyping with HISAT2 and HISAT-genotype. Nature Biotechnology 37, 907–915. 10.1038/s41587-019-0201-4 PMC7605509 31375807

[eraf286-B42] Kinoshita A, Richter R. 2020. Genetic and molecular basis of floral induction in *Arabidopsis thaliana*. Journal of Experimental Botany 71, 2490–2504. 32067033 10.1093/jxb/eraa057 PMC7210760

[eraf286-B43] Lewis HA, Musunuru K, Jensen KB, Edo C, Chen H, Darnell RB, Burley SK. 2000. Sequence-specific RNA binding by a Nova KH domain: implications for paraneoplastic disease and the fragile X syndrome. Cell 100, 323–332. 10676814 10.1016/s0092-8674(00)80668-6

[eraf286-B44] Li D, Liu C, Shen L, Wu Y, Chen H, Robertson M, Helliwell CA, Ito T, Meyerowitz E, Yu H. 2008. A repressor complex governs the integration of flowering signals in Arabidopsis. Developmental Cell 15, 110–120. 18606145 10.1016/j.devcel.2008.05.002

[eraf286-B45] Li Z, Bonaldi K, Uribe F, Pruneda-Paz JL. 2018. A localized *Pseudomonas syringae* infection triggers systemic clock responses in Arabidopsis. Current Biology 28, 630–639. 29398214 10.1016/j.cub.2018.01.001 PMC5820129

[eraf286-B46] Lim M-H, Kim J, Kim Y-S, Chung K-S, Seo Y-H, Lee I, Kim J, Hong CB, Kim H-J, Park C-M. 2004. A new Arabidopsis gene, *FLK*, encodes an RNA binding protein with K homology motifs and regulates flowering time via *FLOWERING LOCUS C*. The Plant Cell 16, 731–740. 14973162 10.1105/tpc.019331 PMC385284

[eraf286-B47] Love MI, Huber W, Anders S. 2014. Moderated estimation of fold change and dispersion for RNA-Seq data with DESeq2. Genome Biology 15, 550. 25516281 10.1186/s13059-014-0550-8 PMC4302049

[eraf286-B48] Lyons R, Iwase A, Gänsewig T, et al 2013. The RNA-binding protein FPA regulates flg22-triggered defense responses and transcription factor activity by alternative polyadenylation. Scientific Reports 3, 2866. 24104185 10.1038/srep02866 PMC3793224

[eraf286-B49] Makeyev A V, Liebhaber SA. 2002. The poly(C)-binding proteins: a multiplicity of functions and a search for mechanisms. RNA 8, 265–278. 12003487 10.1017/s1355838202024627 PMC1370249

[eraf286-B50] Mansour MMF, Hassan FAS. 2022. How salt stress-responsive proteins regulate plant adaptation to saline conditions. Plant Molecular Biology 108, 175–224. 34964081 10.1007/s11103-021-01232-x

[eraf286-B51] Marquardt S, Petrillo E, Manavella PA. 2023. Cotranscriptional RNA processing and modification in plants. The Plant Cell 35, 1654–1670. 36259932 10.1093/plcell/koac309 PMC10226594

[eraf286-B52] Michaels SD, Amasino RM. 1999. *FLOWERING LOCUS C* encodes a novel MADS domain protein that acts as a repressor of flowering. The Plant Cell 11, 949–956. 10330478 10.1105/tpc.11.5.949 PMC144226

[eraf286-B53] Mockler TC, Yu X, Shalitin D, et al 2004. Regulation of flowering time in Arabidopsis by K homology domain proteins. Proceedings of the National Academy of Sciences, USA 101, 12759–12764. 10.1073/pnas.0404552101 PMC515126 15310842

[eraf286-B54] Muñoz-Nortes T, Pérez-Pérez JM, Sarmiento-Mañús R, Candela H, Micol JL. 2017. Deficient glutamate biosynthesis triggers a concerted upregulation of ribosomal protein genes in Arabidopsis. Scientific Reports 7, 6164. 28733652 10.1038/s41598-017-06335-4 PMC5522406

[eraf286-B55] Mur LAJ, Kenton P, Atzorn R, Miersch O, Wasternack C. 2006. The outcomes of concentration-specific interactions between salicylate and jasmonate signaling include synergy, antagonism, and oxidative stress leading to cell death. Plant Physiology 140, 249–262. 16377744 10.1104/pp.105.072348 PMC1326048

[eraf286-B56] Ni B, Klein M, Hossbach B, Feussner K, Hornung E, Herrfurth C, Hamberg M, Feussner I. 2025. *Arabidopsis* GH3.10 conjugates jasmonates. Plant Biology 27, 476–491. 40095511 10.1111/plb.70001 PMC12096059

[eraf286-B57] Nicastro G, Taylor IA, Ramos A. 2015. KH–RNA interactions: back in the groove. Current Opinion in Structural Biology 30, 63–70. 25625331 10.1016/j.sbi.2015.01.002

[eraf286-B58] Olaetxea M, Garnica M, Erro J, Sanz J, Monreal G, Zamarreño AM, García-Mina JM. 2024. The plant growth-promoting effect of an *Ascophyllum nodosum* (L.) extract derives from the interaction of its components and involves salicylic-, auxin- and cytokinin-signaling pathways. Chemical and Biological Technologies in Agriculture 11, 190.

[eraf286-B59] Ortuño-Miquel S, Rodríguez-Cazorla E, Zavala-Gonzalez EA, Martínez-Laborda A, Vera A. 2019. Arabidopsis HUA ENHANCER 4 delays flowering by upregulating the MADS-box repressor genes *FLC* and *MAF4*. Scientific Reports 9, 1478. 30728422 10.1038/s41598-018-38327-3 PMC6365585

[eraf286-B60] Pandey GK, Kanwar P, Singh A, et al 2015. Calcineurin B-like protein-interacting protein kinase CIPK21 regulates osmotic and salt stress responses in Arabidopsis. Plant Physiology 169, 780–792. 26198257 10.1104/pp.15.00623 PMC4577403

[eraf286-B61] Park HJ, Kim WY, Pardo JM, Yun DJ. 2016. Molecular interactions between flowering time and abiotic stress pathways. International Review of Cell and Molecular Biology 327, 371–412. 27692179 10.1016/bs.ircmb.2016.07.001

[eraf286-B62] Parker MT, Knop K, Zacharaki V, et al 2021. Widespread premature transcription termination of *Arabidopsis thaliana* NLR genes by the spen protein FPA. eLife 10, e65537. 33904405 10.7554/eLife.65537 PMC8116057

[eraf286-B63] Pfaffl MW, Horgan GW, Dempfle L. 2002. Relative expression software tool (REST) for group-wise comparison and statistical analysis of relative expression results in real-time PCR. Nucleic Acids Research 30, e36. 11972351 10.1093/nar/30.9.e36 PMC113859

[eraf286-B64] Quiroz S, Yustis JC, Chávez-Hernández EC, Martínez T, de la Paz Sanchez M, Garay-Arroyo A, Álvarez-Buylla ER, García-Ponce B. 2021. Beyond the genetic pathways, flowering regulation complexity in *Arabidopsis thaliana*. International Journal of Molecular Sciences 22, 5716. 34071961 10.3390/ijms22115716 PMC8198774

[eraf286-B65] Ratcliffe OJ, Kumimoto RW, Wong BJ, Riechmann JL. 2003. Analysis of the Arabidopsis MADS AFFECTING FLOWERING gene family: MAF2 prevents vernalization by short periods of cold. The Plant Cell 15, 1159–1169. 12724541 10.1105/tpc.009506 PMC153723

[eraf286-B66] Reddy ASN, Rogers MF, Richardson DN, Hamilton M, Ben-Hur A. 2012. Deciphering the plant splicing code: experimental and computational approaches for predicting alternative splicing and splicing regulatory elements. Frontiers in Plant Science 3, 18. 22645572 10.3389/fpls.2012.00018 PMC3355732

[eraf286-B67] Ripoll JJ, Rodríguez-Cazorla E, González-Reig S, Andújar A, Alonso-Cantabrana H, Perez-Amador MA, Carbonell J, Martínez-Laborda A, Vera A. 2009. Antagonistic interactions between Arabidopsis K-homology domain genes uncover *PEPPER* as a positive regulator of the central floral repressor *FLOWERING LOCUS C*. Developmental Biology 333, 251–262. 19576878 10.1016/j.ydbio.2009.06.035

[eraf286-B68] Ripoll JJ, Bailey LJ, Mai Q-A, Wu SL, Hon CT, Chapman EJ, Ditta GS, Estelle M, Yanofsky MF. 2015. microRNA regulation of fruit growth. Nature Plants 1, 15036. 27247036 10.1038/nplants.2015.36

[eraf286-B69] Rodríguez-Cazorla E, Ripoll JJ, Andújar A, Bailey LJ, Martínez-Laborda A, Yanofsky MF, Vera A. 2015. K-homology nuclear ribonucleoproteins regulate floral organ identity and determinacy in Arabidopsis. PLoS Genetics 11, e1004983. 25658099 10.1371/journal.pgen.1004983 PMC4450054

[eraf286-B70] Rodríguez-Cazorla E, Ortuño-Miquel S, Candela H, Bailey-Steinitz LJ, Yanofsky MF, Martínez-Laborda A, Ripoll J-J, Vera A. 2018. Ovule identity mediated by pre-mRNA processing in Arabidopsis. PLoS Genetics 14, e1007182. 29329291 10.1371/journal.pgen.1007182 PMC5785034

[eraf286-B71] Rodríguez-Cazorla E, Ripoll J, Ortuño-Miquel S, Martínez-Laborda A, Vera A. 2020. Dissection of the Arabidopsis *HUA-PEP* gene activity reveals that ovule fate specification requires restriction of the floral A-function. New Phytologist 227, 1222–1234. 32259283 10.1111/nph.16589

[eraf286-B72] Seo M, Jikumaru Y, Kamiya Y. 2011. Profiling of hormones and related metabolites in seed dormancy and germination studies. Methods in Molecular Biology 773, 99–111. 21898252 10.1007/978-1-61779-231-1_7

[eraf286-B73] Shine M, Gordon J, Schärfen L, Zigackova D, Herzel L, Neugebauer KM. 2024. Co-transcriptional gene regulation in eukaryotes and prokaryotes. Nature Reviews. Molecular Cell Biology 25, 534–554. 38509203 10.1038/s41580-024-00706-2 PMC11199108

[eraf286-B74] Shukla A, Pagán I, Crevillén P, Alonso-Blanco C, García-Arenal F. 2022. A role of the flowering genes in the tolerance of *Arabidopsis thaliana* to cucumber mosaic virus. Molecular Plant Pathology 23, 175–187. 34672409 10.1111/mpp.13151 PMC8743021

[eraf286-B75] Singh V, Roy S, Giri MK, Chaturvedi R, Chowdhury Z, Shah J, Nandi AK. 2013. *Arabidopsis thaliana FLOWERING LOCUS D* is required for systemic acquired resistance. Molecular Plant-Microbe Interactions 26, 1079–1088. 23745676 10.1094/MPMI-04-13-0096-R

[eraf286-B76] Siomi H, Matunis MJ, Michael WM, Dreyfuss G. 1993. The pre-mRNA binding K protein contains a novel evolutionarily conserved motif. Nucleic Acids Research 21, 1193–1198. 8464704 10.1093/nar/21.5.1193 PMC309281

[eraf286-B77] Thatcher LF, Kamphuis LG, Hane JK, Oñate-Sánchez L, Singh KB. 2015. The Arabidopsis KH-domain RNA-binding protein ESR1 functions in components of jasmonate signalling, unlinking growth restraint and resistance to stress. PLoS One 10, e0126978. 25985302 10.1371/journal.pone.0126978 PMC4436139

[eraf286-B78] Thorvaldsdottir H, Robinson JT, Mesirov JP. 2013. Integrative genomics viewer (IGV): high-performance genomics data visualization and exploration. Briefings in Bioinformatics 14, 178–192. 22517427 10.1093/bib/bbs017 PMC3603213

[eraf286-B79] Trapnell C, Hendrickson DG, Sauvageau M, Goff L, Rinn JL, Pachter L. 2013. Differential analysis of gene regulation at transcript resolution with RNA-Seq. Nature Biotechnology 31, 46–53. 10.1038/nbt.2450 PMC3869392 23222703

[eraf286-B80] Veley KM, Michaels SD. 2008. Functional redundancy and new roles for genes of the autonomous floral-promotion pathway. Plant Physiology 147, 682–695. 18408043 10.1104/pp.108.118927 PMC2409018

[eraf286-B81] Wang T, Zhang X. 2021. Genome-wide dynamic network analysis reveals the potential genes for MeJA-induced growth-to-defense transition. BMC Plant Biology 21, 450. 34615468 10.1186/s12870-021-03185-1 PMC8493714

[eraf286-B82] Wang Y, Lv T, Fan T, Zhou Y, Tian C-E. 2025. Research progress on delayed flowering under short-day condition in *Arabidopsis thaliana*. Frontiers Plant Science 16, 1523788. 10.3389/fpls.2025.1523788 PMC11926150 40123949

[eraf286-B83] Wang Y, Schuck S, Wu J, Yang P, Döring AC, Zeier J, Tsuda K. 2018. A MPK3/6–WRKY33–ALD1–pipecolic acid regulatory loop contributes to systemic acquired resistance. The Plant Cell 30, 2480–2494. 30228125 10.1105/tpc.18.00547 PMC6241261

[eraf286-B84] Wasternack C, Hause B. 2013. Jasmonates: biosynthesis, perception, signal transduction and action in plant stress response, growth and development. An update to the 2007 review in *Annals of Botany*. Annals of Botany 111, 1021–1058. 23558912 10.1093/aob/mct067 PMC3662512

[eraf286-B85] Widemann E, Miesch L, Lugan R, Holder E, Heinrich C, Aubert Y, Miesch M, Pinot F, Heitz T. 2013. The amidohydrolases IAR3 and ILL6 contribute to jasmonoyl-isoleucine hormone turnover and generate 12-hydroxyjasmonic acid upon wounding in Arabidopsis leaves. Journal Biological Chemistry 288, 31701–31714. 10.1074/jbc.M113.499228 PMC3814765 24052260

[eraf286-B86] Wu Z, Zhu D, Lin X, et al 2016. RNA-binding proteins at RZ-1B and at RZ-1C play a critical role in regulation of pre-mRNA splicing and gene expression during Arabidopsis development. The Plant Cell 28, 55–73. 26721863 10.1105/tpc.15.00949 PMC4746689

[eraf286-B87] Wu Z, Fang X, Zhu D, Dean C. 2020. Autonomous pathway: *FLOWERING LOCUS C* repression through an antisense-mediated chromatin-silencing mechanism. Plant Physiology 182, 27–37. 31740502 10.1104/pp.19.01009 PMC6945862

[eraf286-B88] Xiong L, Ishitani M, Lee H, Zhu JK. 1999. *HOS5*—a negative regulator of osmotic stress-induced gene expression in *Arabidopsis thaliana*. The Plant Journal 19, 569–578. 10504578 10.1046/j.1365-313x.1999.00558.x

[eraf286-B89] Zavala-Gonzalez EA, Rodríguez-Cazorla E, Escudero N, Aranda-Martinez A, Martínez-Laborda A, Ramírez-Lepe M, Vera A, Lopez-Llorca LV. 2017. *Arabidopsis thaliana* root colonization by the nematophagous fungus *Pochonia chlamydosporia* is modulated by jasmonate signaling and leads to accelerated flowering and improved yield. New Phytologist 213, 351–364. 27456071 10.1111/nph.14106

[eraf286-B90] Zavaliev R, Dong X. 2024. NPR1, a key immune regulator for plant survival under biotic and abiotic stresses. Molecular Cell 84, 131–141. 38103555 10.1016/j.molcel.2023.11.018 PMC10929286

[eraf286-B91] Zeng L, Chen H, Wang Y, Hicks D, Ke H, Pruneda-Paz J, Dehesh K. 2022. ORA47 is a transcriptional regulator of a general stress response hub. The Plant Journal 110, 562–571. 35092704 10.1111/tpj.15688

[eraf286-B93] Zhang N, Zhao B, Fan Z, Yang D, Guo X, Wu Q, Yu B, Zhou S, Wang H. 2020a. Systematic identification of genes associated with plant growth–defense tradeoffs under JA signaling in Arabidopsis. Planta 251, 43. 31907627 10.1007/s00425-019-03335-8

[eraf286-B92] Zhang H, Zhao Y, Zhu JK. 2020b. Thriving under stress: how plants balance growth and the stress response. Developmental Cell 55, 529–543. 33290694 10.1016/j.devcel.2020.10.012

[eraf286-B94] Zhang N, Zhou S, Yang D, Fan Z. 2020c. Revealing shared and distinct genes responding to JA and SA signaling in Arabidopsis by meta-analysis. Frontiers in Plant Science 11, 512053. 10.3389/fpls.2020.00908 PMC7333171 32670328

[eraf286-B95] Zhao H, Wei Z, Shen G, Chen Y, Hao X, Li S, Wang R. 2022. Poly(rC)-binding proteins as pleiotropic regulators in hematopoiesis and hematological malignancy. Frontiers in Oncology 12, 1045797. 36452487 10.3389/fonc.2022.1045797 PMC9701828

[eraf286-B96] Zheng Z, Qamar SA, Chen Z, Mengiste T. 2006. Arabidopsis WRKY33 transcription factor is required for resistance to necrotrophic fungal pathogens. The Plant Journal 48, 592–605. 17059405 10.1111/j.1365-313X.2006.02901.x

