# Supplementary material for: The KH-domain genes FLK and HOS5 integrate flowering and stress responses in Arabidopsis thaliana
Source: J Exp Bot. 2025 Jun 27;76(18):5382–400. doi: 10.1093/jxb/eraf286 (PMC12598764; doi:10.1093/jxb/eraf286)
Supplement: eraf286_Supplementary_Data [file eraf286_supplementary_data.zip › jexbot314878-file002.pdf]

Table S1. Information about molecular genotyping, qRT-PCR, oligonucleotides, and supplementary references.

Purpose	Gene	Name	Oligonucleotide sequence (5'-3')
qRT-PCR	*AP1*	AP1QRT 1F	CTTACGCCGAAAGACAGCTT
		AP1QRT 1R	ATAGGCTGAAGGAAATGGC
	CEJ1	SJ1-333	GCGTTCAAGACTTTGGCTCG
		SJ1-334	CTTTTTCCTGATCGTTGCCGC
	DAP2	SJ1-299	CAACCCCTTGACATCAAAGGTG
		SJ1-300	CTCTAGTGGCGATGTTACTTCC
	ERF103	SJ1-179	GGATCAAAGGCGATTCTGAATTTCC
		SJ1-180	GTCTCTCTTCCGTTTGTTGTACAG
	DREB2A E1-E2	SJ1-459	GCAAGAAGACTAAACACGAAAGC
		SJ1-460	GATTTCCTTTTCCTCGATGTATC
	DREB2A E1-I1	SJ1-459	GCAAGAAGACTAAACACGAAAGC
		SJ1-461	ACCAATTGACGAGACCTAATCAG
	FER	SJ1-305	GTGGTGGTGCTTCTAATCTAAC
		SJ1-306	GAAAACTCTCGCCGTCATGTAA
	FLC E6-E7	SJ1-192	GGCTAGCCAGATGGAGAATAATC
		SJ1-193	TCAACCGCCGATTTAAGGTGG
	FLC I6-E7	SJ1-194	CCTTGGATAGAAGACAAAAAGAGGGAG
		SJ1-195	AGTCACCGGAAGATTG
	GSTF2	SJ1-331	AGCCTTTCCTCTCCCGCAAC
		SJ1-332	AGTCGGTTTGGAGAAGGTTGG
	GSTF6	SJ1-315	CCTTTCATCCTTCGCAACCC
		SJ1-316	CTGGGTCAAACTCATGCGAC
	HOS5 (*hos5-2*)	SJ1-190	GAATCTGAACTTGCAGCAAGTTAG
		SJ1-191	GTGTTCACTTTCACCCGCCTTTG
	HOS5 (*hos5-5*)	KH5-2FF	AAGAGCTTGTGTTCCAGATTCTC
		KH5-2R	TGGAAGCTGGTGGTGTCTC
	HSFB2A E1-E2	SJ1-466	CACAACAATTTCTCTAGTTTCGTTC
		SJ1-467	GGATCTCACGGAGAAGAC
	HSFB2A I1-E2	SJ1-467	GGATCTCACGGAGAAGAC
		SJ1-468	CGATTCCTTCTATTTCCATTGTTC

	JAZ1	SJ1-311	AACTTGGCGAGCAAAGGCAC
		SJ1-312	TTGGGGAGGATTGGATTGGC
	JAZ6	SJ1-307	CTCTACTTCTACCGAAGACAAAG
		SJ1-308	CTTTGTCTTCAGGAAACTCGTTG
	LOX3 E1-E2	SJ1-472	CTGATAAAATTGGTCGAAACATTG
		SJ1-473	CCTTAGTTTTCGATTTCTTTGACC
	LOX3 E1-I1	SJ1-472	CTGATAAAATTGGTCGAAACATTG
		SJ1-474	TACACGATCTTCAGAAAATATGTG
	MPK3	SJ1-427	AGCGATGAAGAAGATAGCTAATG
		SJ1-428	TGAACTGTCTTCTTAGTGGTGG
	PDF1.2	SJ1-267	TAAGTTTGCTTCCATCATCACCC
		SJ1-268	CTTGCATGCATTACTGTTTCCG
	PDF1.2C	SJ1-462	CATCATCACCTTCCTTTTCGC
		SJ1-463	CATGCATTACTGTTTCCGCAAA
	PEN3	SJ1-303	TGCTTTCAACGGTCTAGTAGTC
		SJ1-304	AATGTTGAAGAGGGCTGTGAAAC
	TEM1	SJ1-94	GAGCGACGTCGGGAAGCTGAACCGTTTA
		SJ1-95	TTTAACGAACCGGCTCCAGCCCTTGGT
	WRKY33	SJ1-365	GATATTGACATTCTTGACGACGG
		SJ1-366	GAGCTGCAGGAACATCGTGG
	WRKY40	SJ1-445	CCTCTTTGGTCGATACTTCATTAG
		SJ1-446	CAAGACGTTGTAGTTGTCACAC

Purpose	Name	Oligonucleotide sequence (5'-3')	PCR products size in base pairs
hos5-2 genotyping	SJ1-161	CAGCTACCTCCTCGTTCCATAGAA	1156 wild-type<br>~700 mutant
	SJ1-162	ATGGCGTCAGAAGCTTCGCCTT	
	LBb1.3	AACCAGCGTGGACCGCTTGCTG	
hos5-5 genotyping	SJ1-157	GTTCCAGTTGGTGAGAATGTCTAC	701 wild-type<br>~500 mutant
	SJ1-157	GAGAGCTCCTTGAACATATGACTC	
	LBb1.3	AACCAGCGTGGACCGCTTGCTG	

In qRT-PCR experiments, E and I (with numerals) refer to exons and introns, respectively.

The genotyping procedures for *flc-3* and *flk-2* were as previously reported (Ripoll et al., 2009)

Other primers previously used in qRT-PCR experiments:

ACT2	Serrano *et al.*, 2010
FLC E1-E2	Ripoll et al., 2009
FLC E1-I1	Ripoll et al., 2009
FT	Ripoll et al., 2009
MAF4	Zavala-González *et al.*, 2017
MAF5	Zong *et al.* 2021
OTC	Rodríguez-Cazorla et al., 2015
SOC1	Zavala-González et al., 2017
TSF	Zavala- González et al., 2017

Primers used in Y2H and BiFC cloning experiments were the same as previously reported:

FLK cloning	Rodríguez-Cazorla et al., 2015
CPL1 and *HOS5*/*RCF3* cloning	Rodríguez-Cazorla et al., 2018

Supplementary references

The following references appear in the legend of Figure S6

He, Y., Xu, J., Wang, X., He, X., Wang, Y., Zhou, J., Zhang, S., Meng, X. 2019. The Arabidopsis Pleiotropic Drug Resistance Transporters PEN3 and PDR12 mediate camalexin secretion for resistance to *Botrytis cinerea*. Plant Cell, **31**, 2206.

Jiang, W., Wang, Z., Li, Yali, et al. 2024. *FERONIA* regulates salt tolerance in Arabidopsis by controlling photorespiratory flux. Plant Cell, **36**, 4732–4751.

Lee, S.H., Li, C.W., Koh, K.W., Chuang, H.Y., Chen, Y.R., Lin, C.S., Chan, M.T. 2014. MSRB7 reverses oxidation of GSTF2/3 to confer tolerance of *Arabidopsis thaliana* to oxidative stress. J. Exp. Bot., **65**, 5049–5062.

Lieberherr, D., Wagner, U., Dubuis, P.H., Métraux, J.P., Mauch, F. 2003. The rapid induction of glutathione S-transferases AtGSTF2 and AtGSTF6 by avirulent *Pseudomonas syringae* is the result of combined salicylic acid and ethylene signaling. Plant Cell Physiol., **44**, 750–757.

Maruyama, Y., Yamoto, N., Suzuki, Y., Chiba, Y., Yamazaki, K. ichi, Sato, T., Yamaguchi, J. 2013. The Arabidopsis transcriptional repressor ERF9 participates in resistance against necrotrophic fungi. Plant Sci., **213**, 79–87.

Osnato, M., Castillejo, C., Matías-Hernández, L., Pelaz, S. 2012. *TEMPRANILLO* genes link photoperiod and gibberellin pathways to control flowering in Arabidopsis. Nat. Commun., **3**, 808. https://doi.org/10.1038/ncomms1810

Roy, S., Saxena, S., Sinha, A., Nandi, A.K. 2020. DORMANCY/AUXIN ASSOCIATED FAMILY PROTEIN 2 of *Arabidopsis thaliana* is a negative regulator of local and systemic acquired resistance. J. Plant Res., **133**, 409–417.

Sakuma, Y., Maruyama, K., Qin, F., Osakabe, Y., Shinozaki, K., Yamaguchi-Shinozaki, K. (2006) Dual function of an Arabidopsis transcription factor DREB2A in water-stress-responsive and heat-stress-responsive gene expression. Proc. Natl. Acad. Sci. U. S. A., **103**, 18822–18827.

Serrano M, Hubert DA, Dangl JL, Schulze-Lefert P, Kombrink E. 2010. A chemical screen for suppressors of the avrRpm1-RPM1-dependent hypersensitive cell death response in *Arabidopsis thaliana*. Planta **231**, 1013–1023.

Sewelam, N., Kazan, K., Thomas-Hall, S.R., Kidd, B.N., Manners, J.M., Schenk, P.M. 2013. Ethylene response factor 6 is a regulator of reactive oxygen species signaling in Arabidopsis. PLoS One, **8**. https://doi.org/10.1371/journal.pone.0070289

Shin, S.Y., Park, J.S., Park, H. Bin, Moon, K.B., Kim, H.S., Jeon, J.H., Cho, H.S., Lee, H.J. 2021. *FERONIA* confers resistance to photooxidative stress in Arabidopsis. Front. Plant Sci., **12**. https://doi.org/10.3389/fpls.2021.714938

Tsutsui, T., Kato, W., Asada, Y., et al. 2009. DEAR1, a transcriptional repressor of DREB protein that mediates plant defense and freezing stress responses in Arabidopsis. J. Plant Res., **122**, 633–643.

Vermeirssen, V., Vermeirssen, V., Parys, T. Van, Breusegem, F., Van de Peer, Y. 2014. Arabidopsis ensemble reverse-engineered gene regulatory network discloses interconnected transcription factors in oxidative stress. Plant Cell, **26**, 4656–4679.

Wang, L., Yang, T., Lin, Q., Wang, B., Li, X., Luan, S., Yu, F. 2020. Receptor kinase FERONIA regulates flowering time in Arabidopsis. BMC Plant Biol., **20**. https://doi.org/10.1186/s12870-019-2223-y

Wu, X., Han, Y., Zhu, X., Shah, A., Wang, W., Sheng, Y., Fan, T., Cao, S. 2019. Negative regulation of cadmium tolerance in Arabidopsis by *MMDH2*. Plant Mol. Biol., **101**, 507–516.

Wunderlich, M., Groß-Hardt, R., Schöffl, F. 2014. Heat shock factor HSFB2a involved in gametophyte development of *Arabidopsis thaliana* and its expression is controlled by a heat-inducible long non-coding antisense RNA. Plant Mol. Biol., **85**, 541–550.

Zong W, Zhao B, Xi Y, Bordiya Y, Mun H, Cerda NA, Kim DH, Sung S. 2021. DEK Domain-Containing Proteins Control Flowering Time in Arabidopsis. The New Phytologist **231**, 182–192.
